# Composition-Modulated Strontium Aluminate Phosphors with Continuously Tunable Visible Emission for Advanced Display, Thermometry and Photothermal Conversion

**DOI:** 10.3390/ma19112351

**Published:** 2026-06-02

**Authors:** Jingwen Yang, Guijian Guan

**Affiliations:** 1 Institute of Molecular Plus, Tianjin University, Tianjin 300072, China; yjw0326_@tju.edu.cn; 2 State Key Laboratory of Advanced Papermaking and Paper-Based Materials, South China University of Technology, Guangzhou 510640, China

**Keywords:** strontium aluminate, europium doping, photoluminescence, multicolor emission, optical thermometry, photothermal conversion

## Abstract

**Highlights:**

- A facile composition-regulation strategy via tuning the Al/Sr ratio enables Eu-doped strontium aluminate phosphors with continuous full-visible emission color tuning (red to blue).
- The Al/Sr = 1 phosphor achieves a high absolute quantum yield of 66.2% and acts as a luminescent thermometer with 0.27% K^−1^ relative sensitivity and ~0.005 K resolution.
- Eu-doped strontium aluminate with Al/Sr = 2 exhibits efficient photothermal conversion, reaching ~72.8 °C under 980 nm laser irradiation (1 W·cm^−2^) within 10 s.

**Abstract:**

This study demonstrates a single phosphor material system capable of continuously tuning color across the entire visible spectrum while integrating multiple luminescent functionalities. A series of these phosphors was conveniently synthesized with varying Al/Sr ratios in the reactants, enabling the emission color to progress through red, orange, yellow, green and blue. We systematically investigated the photoluminescence mechanisms by correlating crystal phase evolution with europium ion site occupancy and exploiting the resulting multicolor-emitting phosphors in optical display and anti-counterfeiting demonstrations. The relationships between composition, structure, and luminescence were revealed commendably, alongside more functional evaluations of europium-doped strontium aluminate phosphors. Notably, at an equimolar Al/Sr ratio of 1 (with 2 at% Eu doping), the phosphor achieves a high absolute quantum yield of 66.2% and functions as a luminescent optical thermometer with a relative sensitivity of 0.27% K^−1^ and temperature resolution of ~0.005 K. At a non-equimolar Al/Sr ratio of 2, the Eu-doped phosphor exhibits efficient photothermal conversion, reaching ~72.8 °C under 980 nm laser irradiation (1 W·cm^−2^) within 10 s. This work introduces a facile composition-regulation strategy for designing multicolor-tunable, multifunctional phosphors, highlighting promising applications in optical displays, anti-counterfeiting, luminescence thermometry and photothermal conversion.

## 1. Introduction

Photoluminescent materials—capable of absorbing high-energy light and re-emitting lower-energy light (downconversion) or, conversely, absorbing low-energy light and emitting higher-energy light (upconversion)—have found broad applications in sensors, bioimaging, optical displays, information encryption, anti-counterfeiting and more [1,2,3,4,5,6]. Each application imposes distinct requirements on phosphor properties. For example, advanced display technology demands emitters with narrow emission bandwidths, high color purity, and high quantum yield, while optical thermometry requires materials with excellent thermal stability and high temperature sensitivity, and photothermal conversion relies on efficient light-to-heat conversion capability. More significantly, the above-mentioned three core applications (advanced display, thermometry, photothermal conversion) are increasingly pursuing integration in a single-material system to reduce device fabrication costs and improve applicability [7,8,9,10,11,12]. To date, various photoluminescent materials have been explored, including organic fluorescent dyes, semiconductor quantum dots and inorganic phosphors doped with lanthanide ions [13,14,15]. Organic dyes offer easily tunable chemistry but often suffer from poor photostability and high synthesis costs. Semiconductor quantum dots exhibit toxicity and short luminescence lifetimes, and scaling up their production into solid matrices remains challenging [16,17]. In contrast, inorganic phosphors doped with lanthanide ions combine the advantages of chemical stability with rich electronic energy levels spanning UV to NIR. Such phosphors typically exhibit stable luminescence, readily tunable emission colors, and long excited-state lifetimes, making them among the competitive candidates for advanced photoluminescent applications [18,19,20,21].

Recent efforts have increasingly focused on multicolor and multimodal luminescent phosphors that can serve diverse roles such as anti-counterfeiting and optical sensing. For example, vacuum-synthesized ZnS:Er^3+^ phosphors exhibit multicolor (orange–green) emission and multi-mode readouts for anti-counterfeiting and temperature sensing applications [22]. Similarly, materials like La_4_GeO_8_:Eu^2+^,Er^3+^ can provide dual-mode luminescence detectable under both UV and NIR excitation [23]. Furthermore, phosphors with dual-emission centers (such as codoped vanadates or molybdates) achieve tunable color output across two different excitation modalities, showing potential as dual-mode optical thermometers [24,25]. These examples underscore the demand for phosphors that combine multiple luminescent behaviors in a single host lattice. In recent years, composition modulation (e.g., adjusting the ratio of matrix elements) has emerged as a novel and effective strategy for tuning luminescent properties, which has the advantages of higher stability, wider tuning range, and simpler preparation process compared with traditional dopant-type/concentration adjustment methods [26,27,28]. However, most of the existing studies on color-tunable phosphors still rely on changing the dopant type or concentration within a given host, and studies on achieving continuous emission tuning across the entire visible spectrum through composition modulation of the host itself are very rare. More importantly, there are few reports on integrating advanced display, thermometry and photothermal conversion functions in a single strontium aluminate system, which severely limits the practical application of this excellent host material in multi-field scenarios.

Strontium aluminate has emerged as an excellent host matrix for lanthanide doping due to its high thermal stability and flexible structural diversity. By adjusting the stoichiometric Al/Sr ratio, strontium aluminate forms multiple crystalline phases (e.g., Sr_3_Al_2_O_6_, SrAl_2_O_4_, Sr_4_Al_14_O_25_), each offering a unique lattice environment. Importantly, the Sr^2+^ cation sites in these lattices vary in coordination number and symmetry, providing a natural platform for tuning the luminescent behavior of doped activator ions [29,30]. Europium (Eu) is especially suitable for doping into Sr-based aluminates because the ionic radius of Eu^2+^ (≈1.20 Å) closely matches that of Sr^2+^ (≈1.18 Å) [31]. As a result, Eu ions can substitute into Sr sites with relative ease, often leading to the presence of both Eu^2+^ and Eu^3+^ in the lattice (Eu^2+^ can form via in situ reduction of Eu^3+^ under a reducing condition). The coexistence of Eu^2+^ and Eu^3+^ in a single host is highly advantageous: Eu^2+^ typically emits broad-band blue–green light (allowed 4f^6^5d^1^ → 4f^7^ transitions), while Eu^3+^ emits narrow-band orange–red light (forbidden ^5^D_0_ → ^7^F_j_ transitions) [25,32]. This unique luminescent behavior of Eu-doped strontium aluminate, combined with the adjustable crystal phase via Al/Sr ratio regulation, lays a solid foundation for realizing continuous visible emission tuning and integrating multiple functions (display, thermometry, photothermal conversion) in a single-material system. By controlling the crystal phase and Eu site occupancy, one can leverage both valence states to achieve tunable multicolor emission within one material system.

Previous studies on color-tunable phosphors have mostly relied on changing the dopant type or concentration within a given host. However, continuous tunability spanning the entire visible spectrum in the same host system has rarely been reported. This gap has limited the development of single materials for multifunctional applications such as full-color displays, optical thermometry and photothermal conversion. Herein, we address this challenge by developing a composition-regulated strategy: we synthesize a family of Eu-doped strontium aluminate phosphors by systematically varying the Al/Sr ratio (*x*) in the precursors. By this simple high-temperature solid-state method, we obtain phosphors with *x* = 1/2, 1, 3/2, 2, 3, and 4, corresponding to different dominant crystal phases. These phases can incorporate Eu ions as activators, yielding multicolored emissions without changing the host material. We thoroughly characterize the photoluminescence (PL) and photophysical properties of the prepared phosphors. As a result, we demonstrate a single-material platform in which the emission wavelength can be tuned across the visible range (red to blue) solely by adjusting the host composition while also enabling multiple functionalities, including optical display, anti-counterfeiting, luminescent thermometry and photothermal conversion. The significance of this work lies in its contribution to research on continuous visible emission tuning in composition-modulated strontium aluminate phosphors, providing a simple and scalable preparation strategy for multifunctional luminescent materials and offering a feasible approach to promote the practical application of strontium aluminate phosphors in advanced display, thermometry and photothermal conversion fields.

## 2. Experimental Section

### 2.1. Materials

Strontium carbonate (SrCO_3_, 99.95%), aluminum oxide (Al_2_O_3_, 99.99%) and europium oxide (Eu_2_O_3_, 99.99%) were purchased from Aladdin Ltd., Shanghai, China. Ethanol (C_2_H_5_OH, analytical grade) and polyvinylpyrrolidone K30 (PVP, M*w* ≈ 40,000) were obtained from Innochem, Beijing, China. Boric acid (H_3_BO_3_, 99.5%) was purchased from Saitong Chemical, Beijing, China. Phenolic epoxy resin (formula C_21_H_25_NO_4_) was purchased from Zhonghe Shengtai Chemical Co., Tianjin, China. Isopropyl alcohol (C_3_H_8_O, 99.5%) and (3-aminopropyl)triethoxysilane (APTES, C_9_H_23_NO_3_Si, 99%) were obtained from Macklin and Rhawn, Shanghai, China. A forming gas mixture of 95% N_2_/5% H_2_ was supplied by Liufang Industrial Gas, Tianjin, China.

### 2.2. Synthesis of Eu-Doped Strontium Aluminate Phosphors

A series of Eu-doped strontium aluminate phosphors was synthesized via a conventional high-temperature solid-state route by adjusting the Al/Sr ratios in the reactants. Stoichiometric amounts of Al_2_O_3_ and SrCO_3_ were weighed to achieve molar ratios of Al/Sr (*x* = 1/2, 1, 3/2, 2, 3, 4). To each mixture, Eu_2_O_3_ was added at a fixed doping level of 2 at% Eu (relative to Sr in atomic percent), and H_3_BO_3_ (5 at%) was added as a flux. The powders were thoroughly mixed and ground. For efficient homogenization, the mixture (placed in a 10 mL agate milling jar with zirconia beads; mass ratio of beads:powers ≈ 15:1) was ball-milled at 40 Hz for 10 min. The resultant precursor powder was loaded into an alumina crucible and calcined in a tube furnace under a reducing atmosphere (95% N_2_/5% H_2_), which facilitated the controlled reduction of Eu^3+^ to Eu^2+^ under varying ratios of Al/Sr in reactants during the following solid-state reaction. The furnace was evacuated to ~100 Pa, then purged with the N_2_/H_2_ gas mix (this evacuation–purge cycle was repeated twice to ensure an oxygen-poor environment). The sample was heated to 800 °C at 10 °C/min, then to 1200 °C at 5 °C/min and held at 1200 °C for 2 h before being cooled to room temperature. The obtained cake was lightly ground to yield fine Eu-doped strontium aluminate phosphor powders.

Using the same procedure, we also optimized the Eu dopant concentration and flux amount for one selected composition. We prepared a series of samples with Eu doping concentrations of 1/2, 1, 2, 3, 4 and 8 at% to determine the optimal activator level. Similarly, the flux (H_3_BO_3_) concentration was varied from 0 to 10 at% (0, 1, 2, 5 and 10 at%) to optimize crystal growth and luminescence.

### 2.3. Preparation of Luminescent Inks

To demonstrate the use of these phosphors in luminescent displays (e.g., security printing), a phosphor-based ink was formulated using an epoxy resin as the carrier. Surface modification of the phosphor particles was first performed to improve their dispersibility in the epoxy solution. In a typical procedure, 0.2 g of Eu-doped strontium aluminate powders was mixed with 2 mL of ethanol in a 5 mL milling vial containing zirconia beads and wet-milled at 30 Hz for 1 h to break up agglomerates and obtain a uniform particle size distribution. The suspension was transferred to another vial containing 2 mL ethanol and 0.02 g PVP, then stirred for 40 min. During this process, PVP molecules adsorbed onto the particle surfaces, forming a polymer coating that enhanced compatibility with the epoxy matrix. The PVP-modified phosphor was collected by centrifugation (4000 rpm, 3 min) and dried.

In parallel, a functional epoxy resin solution was prepared as the ink carrier. First, 2.5 g of the phenolic epoxy resin was combined with 1 mL of isopropanol and stirred at 40 °C for 20 min to obtain a uniform, transparent solution. Next, 1.43 mL of (3-aminopropyl)triethoxysilane (APTES) was added dropwise in three portions (to moderate the exothermic reaction), and the mixture was stirred at room temperature for 1 h. In this step, the amine groups of APTES reacted with epoxy groups, partially curing the resin and introducing silane functionality, yielding a stable, slightly viscous epoxy-based carrier fluid. Finally, the luminescent ink was formulated by blending the PVP-modified phosphor into the epoxy carrier fluid at a solid loading of 20 wt%. The mixture was stirred mechanically for 30 min to ensure homogeneous dispersion of phosphor particles. The resulting composite ink has good uniformity and wettability, allowing it to be readily applied by a paintbrush or pen for writing and drawing. Once applied to a substrate and dried, the patterns written with this ink remain transparent under normal light but exhibit visible luminescent colors under UV excitation.

### 2.4. Measurements of Photothermal Conversion

The photothermal conversion performance of selected phosphors was evaluated by measuring their temperature rise under various illumination sources. Approximately 0.10 g of phosphor powder was packed into a shallow quartz sample holder (10 × 10 mm^2^ area, 1 mm depth) to create a flat sample surface. The sample holder was placed on a thermally insulating stand, and a consistent geometry was maintained with the excitation source: the light source (laser or lamp) was fixed at 5 cm above the sample, normal to its surface. Three irradiation conditions were tested, each with a power density of 1 W·cm^−2^: a continuous 980 nm diode laser (for NIR excitation), a continuous 808 nm diode laser, and simulated sunlight from a Xe arc lamp (with an AM 1.5G filter by Microenerg in Beijing, China). During irradiation, an infrared thermal imaging camera (model HM-TP31-3AUF by Hangzhou Microimage Software Co., Ltd. in China) was used to record the surface temperature of the phosphor at regular time intervals (typically up to 120–180 s). Control measurements (without phosphor) were performed for each light source to account for ambient heating; the net temperature rise of the phosphor was obtained by subtracting the temperature increase of the control. Infrared thermal images were also captured at the end of each irradiation period to visualize the heat distribution.

### 2.5. Characterization

Powder X-ray diffraction (XRD) patterns were recorded on a Rigaku D8 Advance diffractometer (Cu Kα radiation, λ = 1.5406 Å) by Rigaku, Japan. Data were collected from 2θ = 10° to 70° at a scan rate of 20°·min^−1^. The identified crystal phases were matched to standard PDF reference patterns. Transmission electron microscopy (TEM) and energy-dispersive X-ray spectroscopy (EDS) to examine particle morphology and elemental distribution were performed on a Talos X instrument by Thermo Fisher, USA. Ultraviolet–visible diffuse reflectance (UV-Vis DR) spectra were measured with a scanning interval of 1 nm using SolidSpec-3700i spectrophotometer (integrating sphere accessory) by Shimadzu, Japan. The absorption edge and bandgap of samples were analyzed via Tauc plots constructed from the Kubelka–Munk function.

Photoluminescence emission (PL) spectra, photoluminescence excitation (PLE) spectra, and photoluminescence decay curves were obtained with a QuantaMaster 8000 fluorescence spectrometer by HORIBA Scientific, Canada. A 75 W xenon arc lamp was used as the excitation source for steady-state PL and PLE measurements. Emission and excitation slit widths were typically 1 nm with a 0.1 s integration time. Absolute quantum yield (AQY) measurements were carried out on the same spectrometer equipped with an integrating sphere; for each phosphor, the emission (fluorescence) and excitation spectra were recorded under identical conditions, and the AQY was calculated as the ratio of emitted photon count to absorbed photon count (accounting for the baseline contribution from the solvent or matrix). Temperature-dependent PL spectra were collected by mounting the sample on a temperature-controlled heating stage (TAP-02) integrated with the spectrometer by Tianjin Orient KOJI Instrument Co., Ltd. in China. The sample was heated from 300 K to the target temperature with 10 min equilibration at each step, then the PL spectrum was recorded. For each temperature, the measurement was repeated 10 times to ensure reliability.

## 3. Results and Discussion

### 3.1. Composition-Dependent Crystal Structure and Photoluminescence

**Figure 1:**
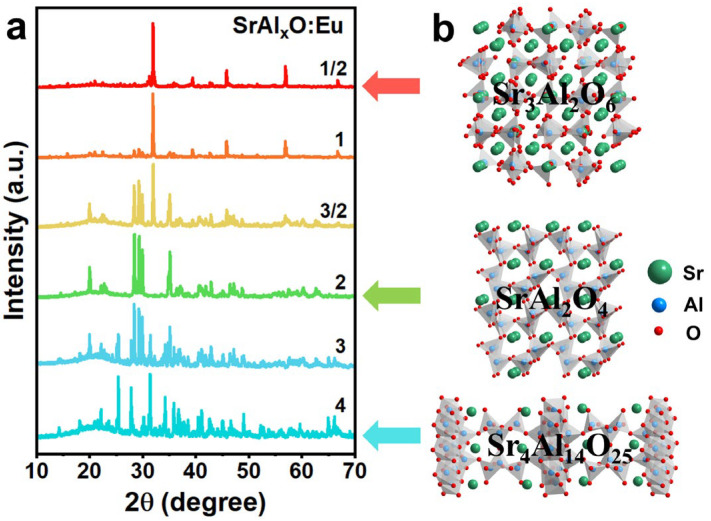
Composition-regulated crystal phases of Eu-doped strontium aluminate phosphors synthesized via high-temperature solid-state reaction. (**a**) XRD patterns of Eu-doped strontium aluminate phosphors (x = 1/2–4) demonstrating phase evolution from Sr_3_Al_2_O_6_ to SrAl_2_O_4_ and Sr_4_Al_14_O_25_ with increasing Al/Sr ratio. (**b**) Corresponding structural motifs of Sr_3_Al_2_O_6_, SrAl_2_O_4_ and Sr_4_Al_14_O_25_ illustrating changes in [AlO_4_] and [AlO_6_] coordination environments.

Strontium aluminate’s phase and structure can be systematically tuned by the Al/Sr ratio (*x*), which, in turn, influences Eu incorporation and luminescence. To validate this, we synthesized a series of Eu-doped strontium aluminate phosphors (with Al/Sr ratios *x* = 1/2–4 in the reactants, 2 at% Eu doping) and characterized their crystal structures by XRD. As shown in Figure 1a, changing the Al/Sr ratio causes an evolution of the crystalline phase. The *x* = 1/2 sample’s diffraction peaks match the cubic-phase Sr_3_Al_2_O_6_ (Figure S1a). In cubic Sr_3_Al_2_O_6_ (space group *Pa*3), Al^3+^ ions are tetrahedrally coordinated by O^2−^ (forming [AlO_4_]^5−^ units) [33]. The *x* = 2 composition yields diffraction peaks matching monoclinic SrAl_2_O_4_ (Figure S1b). Monoclinic SrAl_2_O_4_ (space group *P*2_1_) consists of vertex-sharing [AlO_4_]^5−^ tetrahedra forming six-membered rings, with Sr^2+^ ions situated in the ring cavities. The *x* = 4 sample’s pattern matches orthorhombic Sr_4_Al_14_O_25_ (Figure S1c). Orthorhombic Sr_4_Al_14_O_25_ (space group *Pmma*) features alternating layers of [AlO_6_]^9−^ octahedra and double layers of [AlO_4_]^5−^ tetrahedra [34]. In intermediate compositions, mixed phases occur: for *x* = 1 and 3/2, the XRD reveals a two-phase mixture of Sr_3_Al_2_O_6_ and SrAl_2_O_4_; for *x* = 3, a mixture of SrAl_2_O_4_ and Sr_4_Al_14_O_25_ is observed. These results demonstrate that by adjusting the Al/Sr ratio in the precursor, one can effectively control the crystalline phase of Eu-doped strontium aluminate phosphors.

**Figure 2:**
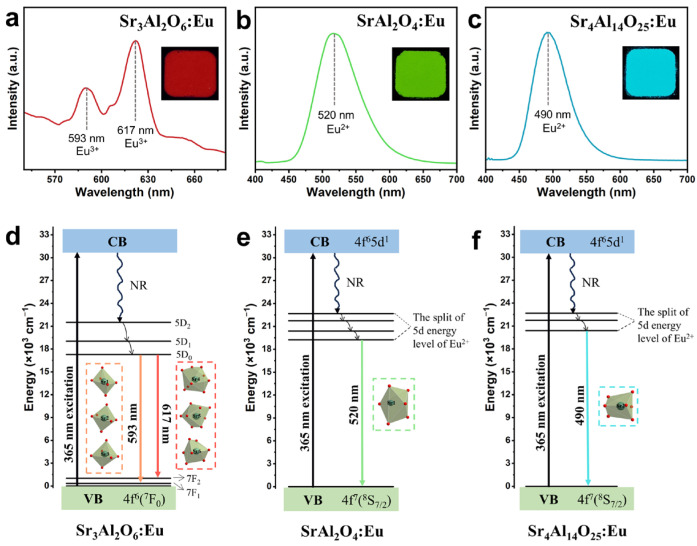
Photoluminescence of Eu-doped strontium aluminate phosphors synthesized via high-temperature solid-state reaction. (**a**–**c**) PL spectra at 365 nm excitation of (**a**) Sr_3_Al_2_O_6_:Eu, (**b**) SrAl_2_O_4_:Eu and (**c**) Sr_4_Al_14_O_25_:Eu, confirming host-regulated emission spanning the red–green–blue range. The insets are photographs of the phosphor powders under 365 nm excitation, showing tunable emission color. (**d**–**f**) Schematic energy transfer diagrams of (**d**) Sr_3_Al_2_O_6_:Eu, (**e**) SrAl_2_O_4_:Eu and (**f**) Sr_4_Al_14_O_25_:Eu. The arrows indicate the transition behaviour of photons between energy levels. The distinct Sr^2+^ coordination environments in different hosts determine Eu site occupation and emission behavior.

Figure 1b presents the crystal structures of the three end-member phases (Sr_3_Al_2_O_6_, SrAl_2_O_4_, Sr_4_Al_14_O_25_) for visual comparison. The ability to host Eu in these lattices without altering the phase is evidenced by the fact that doping ~2 at% Eu^3+^ did not produce any secondary phase peaks in XRD (the patterns of Eu-doped samples align with the undoped standards; see Figure S1). However, Eu doping strongly influences the photoluminescent properties of each phase. Table S1 lists the ionic radii of relevant cations: Eu^2+^ (1.20 Å) is almost the same size as Sr^2+^ (1.18 Å), while Eu^3+^ is smaller (0.95 Å). This size matching means Eu^2+^ will preferentially occupy Sr^2+^ sites in the lattice. Under UV excitation, Eu dopants can be excited (Eu^3+^ through charge-transfer bands or f–f transitions; Eu^2+^ through 4f–5d transitions) and then emit light upon relaxation. The inserted photographs in Figure 2a show representative emission colors of three phosphors (*x* = 1/2, 2, 4) under a 365 nm UV lamp. The Sr_3_Al_2_O_6_:Eu sample (*x* = 1/2) glows red, SrAl_2_O_4_:Eu (*x* = 2) glows green and Sr_4_Al_14_O_25_:Eu (*x* = 4) glows blue. Therefore, by changing *x*, we achieve a continuous progression of emission hue from red to green to blue in this series. The corresponding PL emission spectra (Figure 2a) were recorded under 365 nm excitation for those three samples. Consistent with the observed colors, Sr_3_Al_2_O_6_:Eu (*x* = 1/2) shows dominant emission peaks in the orange–red region (~593 nm and 617 nm, characteristic of Eu^3+ 5^D_0_ → ^7^F_1_ and ^5^D_0_ → ^7^F_2_ transitions, respectively). SrAl_2_O_4_:Eu (*x* = 2) exhibits a broad green emission band peaking at ~520 nm, typical of Eu^2+^ 4f^6^5d^1^ → 4f^7^ transitions in that host. Sr_4_Al_14_O_25_:Eu (*x* = 4) shows a broad blue–green emission peaking at ~490 nm (Eu^2+^ emission in that host). A clear blue shift of the dominant emission is evident as *x* increases: roughly 617 nm (red) → 520 nm (green) → 490 nm (blue). We also measured the absolute quantum yield (AQY) of these samples. Using an integrating sphere, the AQYs for Sr_3_Al_2_O_6_:Eu, SrAl_2_O_4_:Eu and Sr_4_Al_14_O_25_:Eu were determined to be 9.7%, 46.5% and 29.9%, respectively (Figure S2 and Table S2). Thus, the SrAl_2_O_4_:Eu (*x* = 2) composition achieves a notably higher efficiency under 365 nm excitation than the other phases.

To understand the origin of emission color differences, we focus on Eu valence, Sr site occupancy, and local crystal-field effects, which together determine the luminescence mechanism. Figure 2d–f schematically illustrates the luminescence processes in the three host phases. Eu^3+^ emission is strongly governed by local site symmetry. In sites with inversion symmetry (Sr1, Sr2, Sr3), the magnetic-dipole ^5^D_0_ → ^7^F_1_ (orange) transition dominates; in non-centrosymmetric sites (Sr4, Sr5, Sr6), the electric dipole ^5^D_0_ → ^7^F_2_ transition (red) is significantly enhanced [35]. The observed mix of orange (~593 nm) and red (~617 nm) in Sr_3_Al_2_O_6_:Eu thus indicates Eu^3+^ distribution over multiple sites. For Eu^2+^, emission originates from 4f^6^ 5d^1^ → 4f^7^ transitions and is controlled by crystal-field splitting. In SrAl_2_O_4_ (Figure 2e), there are two inequivalent Sr^2+^ sites: one six-coordinated (Sr1, average Sr-O ~2.70 Å) and one seven-coordinated (Sr2, ~2.67 Å). The dominant green emission at ~520 nm is attributed to Eu^2+^ occupying the six-coordinate Sr1 site [36]. In Sr_4_Al_14_O_25_ (Figure 2f), there are also two Sr sites: one ten-coordinated (Sr1, average Sr–O ~2.77 Å) and one seven-coordinated (Sr2, ~2.62 Å). The shorter Sr–O bonds at Sr2 create a stronger crystal field, splitting the Eu^2+^ 5d levels more and leading to a lower-energy emission [37]. The emission at ~490 nm (blue–green) is contributed by the Sr2 site. In short, the Al/Sr ratio controls crystal phase, Sr site geometry, and Eu valence/occupation, enabling continuous red–green–blue emission tuning.

**Figure 3:**
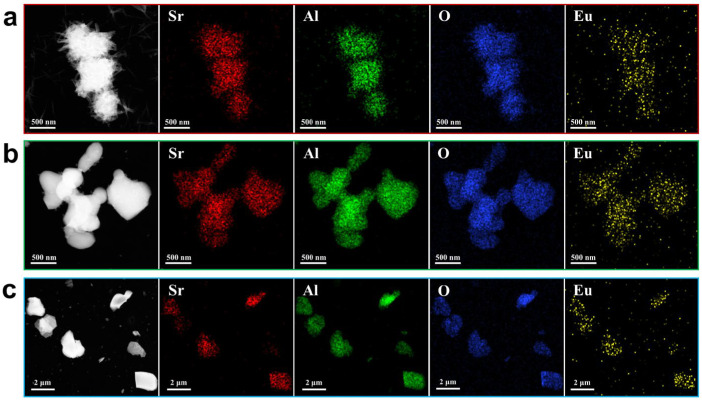
Morphology and compositional uniformity of Eu-doped strontium aluminate phosphors. TEM images indicate nano-needle morphology for (**a**) Sr_3_Al_2_O_6_:Eu, granular particles for (**b**) SrAl_2_O_4_:Eu and block-like grains for (**c**) Sr_4_Al_14_O_25_:Eu. EDS elemental mapping confirms a homogeneous distribution of Eu dopant across each host lattice.

Beyond crystal structure, morphology and composition uniformity can also impact luminescence. Figure 3 shows TEM images and EDS elemental mapping for representative phosphor particles from three samples (*x* = 1/2, 2, 4). As seen in Figure 3 and Figure S3, all samples consist of irregularly shaped grains primarily 1–3 µm in size. The Sr_3_Al_2_O_6_:Eu (*x* = 1/2) sample exhibits a needle-like morphology, with the phosphor particles aggregated into this form [38]. Furthermore, the SrAl_2_O_4_:Eu (*x* = 2) sample is mainly composed of particles with a size less than 1 μm, and the Sr_4_Al_14_O_25_:Eu sample exhibits an angular blocky morphology with an increased particle size compared to SrAl_2_O_4_:Eu. Despite these morphological differences, the EDS maps confirm that Eu is uniformly distributed in all the phosphor particles, with no evidence of Eu-rich clusters or phase segregation. The measured Al/Sr ratios from EDS align closely with the intended stoichiometries (Figure S4), indicating good compositional control. In summary, composition tuning of the strontium aluminate host leads to distinct crystal phases and Eu site environments, which are the key to achieving a wide range of emission colors in this system.

### 3.2. Multicolored Luminescent Display and Anti-Counterfeiting Applications

**Figure 4:**
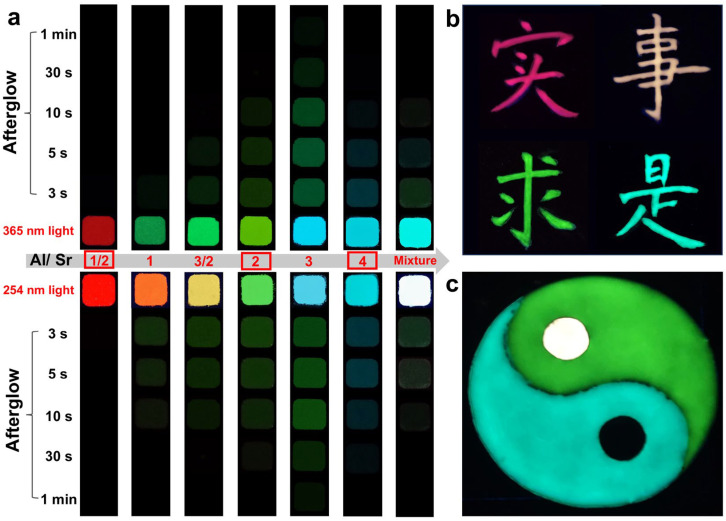
Multicolored luminescent and afterglow behavior enabling anti-counterfeiting applications. (**a**) Luminescent and afterglow images of Eu-doped strontium aluminate phosphors (*x* = 1/2–4) under 254 nm and 365 nm excitation. (**b**) Chinese characters (shí shì qíu shì) written using epoxy-based luminescent ink containing composition-tuned phosphors: under 254 nm, each character emits its corresponding distinct color. (**c**) Tai Chi pattern drawn with different phosphor inks, demonstrating excitation-dependent color switching and persistent luminescence.

With multicolor emission spanning red to blue available from the Eu-doped strontium aluminate series, we next demonstrate their use in luminescent displays for information encoding. Figure 4a provides an overview of the photoluminescence behavior of the full series under two UV excitation wavelengths (254 nm and 365 nm). The bottom row of panel shows the emission from Eu-doped strontium aluminate powders (*x* = 1/2 through 4) under 254 nm excitation, where a smooth progression of color is observed: as *x* increases from 1/2 to 4, the emission color shifts from red → orange → yellow → green → cyan → blue. Under 365 nm excitation (top row of Figure 4a), most compositions emit the same color or slightly dimmer (since 365 nm primarily excites Eu^2+^), but interestingly, the *x* = 1 and 3/2 samples appeared orange–yellow under 254 nm, compared with their green emission under 365 nm. This indicates their mixed Eu^2+^/Eu^3+^ character (as discussed in the next section). Additionally, by combining multiple phosphors, we can create white or custom colors: for example, a mixture of Eu-doped strontium aluminate (*x* = 1/2, 2, 4) phosphors in a 22:1:1 ratio yields a near-white emission under 254 nm (due to the blend of red, green, and blue), while the same mixture appears blue under 365 nm (since the Eu^2+^ emission dominates). Among all samples, the Eu-doped strontium aluminate (*x* = 3) phosphor exhibited the longest afterglow (persistent phosphorescence) after the UV lamp was turned off, indicating excellent trap-mediated long decay, likely due to its mixed-phase nature (SrAl_2_O_4_ + Sr_4_Al_14_O_25_), which can harbor effective trap centers [20,39]. The phosphorescence decay curves of the Eu-doped strontium aluminate phosphors (*x* = 1/2–4), as shown in Figure S5, are calculated by fitting a triple-exponential function, consistent with the decay trend recorded in Figure 4a. The fitted parameters of the phosphorescence decay curves are recorded in Table S3. Among these phosphors, the Eu-doped strontium aluminate phosphor (*x* = 3) exhibits the longest afterglow time.

The rich luminescent colors of the *x* = 1/2–4 phosphors provide an ideal material platform for exploiting luminescent display and anti-counterfeiting applications. To this end, we developed a luminescent ink using the phosphors as the luminescent filler. First, an epoxy–amine resin carrier solution was prepared by reacting the phenolic epoxy resin with (3-aminopropyl)triethoxysilane, as shown in Figure S6a. Then, the luminescent ink was obtained by homogenously blending the PVP-modified phosphors into the epoxy resin carrier solution (at 20 wt% phosphor), as depicted in Figure S6b. Owing to good uniformity and wettability, the resultant luminescent ink was readily applied with a paintbrush or pen for writing and drawing. Figure 4b,c show two practical demonstrations of multicolor phosphor inks. In Figure 4b, four Chinese characters (representing the motto of Tianjin University) were painted on paper using epoxy inks loaded with *x* = 1/2 (red), 3/2 (yellow), 2 (green) and 4 (blue) phosphors. Under 254 nm UV illumination, the characters clearly emit in four different colors, corresponding to the chosen phosphor (the image was taken in a dark environment to emphasize the luminescence). In Figure 4c, a Tai Chi diagram was drawn using inks with *x* = 2 (green), *x* = 4 (blue) and the white-emitting mixture phosphor. Under 254 nm excitation, the Tai Chi pattern is visible in multiple colors as intended. Intriguingly, when switching to 365 nm excitation, the diagram’s appearance changes because each region’s emission color (or brightness) is different at the longer wavelength: for instance, the region painted with the white-mixture phosphor emits blue under 365 nm instead of white. Additionally, the persistence (afterglow) of each phosphor differs; the region with the *x* = 3 phosphor continues to glow the longest time after the UV is turned off. These features could be utilized for multi-layered security: information could be embedded not only in static colors but also in how the image evolves under different excitation wavelengths and in afterglow mode.

Overall, the rich palette of luminescent colors and afterglow behaviors offered by Eu-doped strontium aluminate phosphors provides an appealing platform for anti-counterfeiting inks and dynamic optical tags. The distinct response of each phosphor to different UV wavelengths (254 nm vs. 365 nm) and the presence of persistent luminescence (notably in *x* = 3) mean that multiple authentication steps can be integrated. For example, a security label could be verified by observing one pattern under a 254 nm lamp and a hidden pattern under a 365 nm lamp, as well as by checking for afterglow—features that are difficult to counterfeit with a single type of material. In the next section, we delve deeper into the photophysical properties underlying these behaviors, including excitation-dependent emission and the role of Eu valence states.

### 3.3. Spectroscopic Analysis and Excitation-Dependent Emission Behavior

The initial observations above suggest that some Eu-doped strontium aluminate compositions (especially those around *x* ~1–3/2) exhibit excitation wavelength-dependent luminescence. We investigate this phenomenon in detail here, alongside optimizing dopant and flux levels for the best performance. First, we determined the optimal Eu doping concentration in the SrAl_2_O_4_ host (*x* = 2, which gave the highest AQY among pure phases). Figure S7 shows the PL emission intensity of SrAl_2_O_4_:Eu as a function of Eu doping (1/2–8 at%) under 254 nm excitation. The emission peak position remains ~520 nm for all doping levels (indicating the emission mechanism is unchanged), but the intensity increases with Eu content up to 2 at% and then decreases due to concentration quenching. Thus, 2 at% Eu is optimal for SrAl_2_O_4_, and we fixed 2 at% Eu for all Eu-doped strontium aluminate samples in this study. Similarly, we found that adding a small amount of H_3_BO_3_ flux improved crystallinity and luminescence, with an optimum at ~5 at% flux. Figure S8 shows that 5 at% H_3_BO_3_ yields the highest PL intensity for SrAl_2_O_4_:Eu, whereas 0% or 10 at% flux results in weaker emission (too little flux yields incomplete crystallization; too much causes particle growth and reduced emission) [39]. Therefore, in our final samples, we used 5 at% H_3_BO_3_ as a flux.

**Figure 5:**
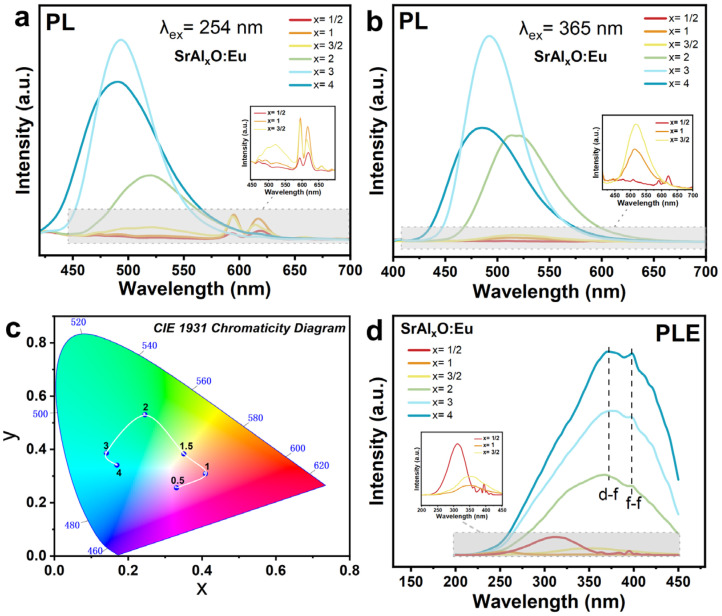
Photoluminescence spectra of Eu-doped strontium aluminate phosphors (*x* = 1/2–4) illustrating the composition-dependent Eu^2+^/Eu^3+^ emission. (**a**,**b**) PL spectra under 254 nm and 365 nm excitation: Eu^3+ 5^D_0_ → ^7^F_1_ (~593 nm) and ^5^D_0_ → ^7^F_2_ (~617 nm) dominate at low Al/Sr ratios, while broad Eu^2+^ 4f^6^5d^1^ → 4f^7^ bands emerge near 520–490 nm at higher ratios; (**c**) CIE chromaticity coordinates revealing continuous color evolution across visible region via Al/Sr tuning under 254 nm excitation; (**d**) PLE spectra corroborating site-dependent energy levels of Eu activators.

With these optimized conditions, we recorded detailed PL and PLE spectra for all compositions. Figure 5a compares the PL spectra of Eu-doped strontium aluminate phosphors (*x* = 1/2–4) under 254 nm excitation (which primarily excites Eu^3+^ through charge transfer or higher 4f levels). For *x* = 1/2, the emission consists of the Eu^3+^ lines at ~593 nm and ~617 nm (orange and red). As *x* increases to 1, the 593 nm (orange) line intensifies markedly, indicating a strong Eu^3+ 5^D_0_ → ^7^F_1_ emission, while the 617 nm red line grows only modestly. At *x* = 3/2, a broad green band (~520 nm) attributed to Eu^2+^ appears and becomes dominant for *x* = 2. At *x* = 3, the Eu^2+^ emission shifts toward ~490 nm (blue–green) and reaches maximum intensity; at *x* = 4, the intensity drops slightly, and the peak remains ~490 nm (consistent with the Sr_4_Al_14_O_25_ host). In summary, under 254 nm excitation, the emission evolves from primarily Eu^3+^ (red/orange) in Al-poor phases to predominantly Eu^2+^ (green/blue) in Al-rich phases.

By contrast, Figure 5b shows the PL spectra under 365 nm excitation (which selectively excites Eu^2+^ because 365 nm photons match Eu^2+^ 4f–5d absorption but are too low in energy to significantly excite Eu^3+^ from the ground state). Under 365 nm, the *x* ≤ 3/2 samples exhibit very weak emissions (since Eu^3+^ cannot be effectively excited and the Eu^2+^ concentration is low in these samples). As *x* increases beyond 3/2, the Eu^2+^ emission grows; the brightest sample under 365 nm is again *x* = 3, with strong ~490 nm emission. Notably, the spectra for *x* = 3 and 4 under 365 nm look similar to those under 254 nm (dominated by Eu^2+^ blue emission), whereas the spectra for *x* = 1 and 3/2 differ drastically between 254 nm and 365 nm.

For example, the *x* = 1 phosphor emitted mainly orange–red (Eu^3+^) under 254 nm but emitted green (Eu^2+^) under 365 nm, which explains the color change observed in Figure 4a. This dual behavior is due to the presence of mixed-valence Eu in the phosphors: 254 nm light excites Eu^3+^ (via O^2−^ → Eu^3+^ charge-transfer band around 250–270 nm), leading to orange–red emission [35], whereas 365 nm light directly excites Eu^2+^ (4f^7^ → 4f^6^5d^1^), yielding green Eu^2+^ emission. For *x* = 3/2, a similar but less pronounced excitation-dependent color shift is seen (from yellowish under 254 nm to greener under 365 nm), indicating that both Eu^3+^ and Eu^2+^ contribute. To quantify the emission colors, we calculated the Commission Internationale de l’Éclairage (CIE) chromaticity coordinates for each composition’s emission under 254 nm [40]. Figure 5c plots these coordinates on a standard CIE 1931 diagram. The trajectory clearly moves through red, orange, yellow, green to blue as *x* increases. The specific (x,y) values are listed in Table S4. This confirms that continuous color tuning has been achieved in this series, a critical property for applications in display and lighting technologies.

To understand the luminescence more scientifically, we measured the PLE spectra (Figure 5d) and PL spectra (Figure S9) of the phosphors at the optimal emission and excitation wavelengths, respectively. For *x* = 1/2, a broad excitation band peaking at ~312 nm is observed (this is an Eu^3+^ charge-transfer band; O^2−^ → Eu^3+^) [35,41]. As *x* increases to 1 and 3/2, the optimal excitation shifts to longer UV (~350–355 nm). For *x* = 2, 3 and 4, the excitation peaks are around 365 nm and 374 nm, which correspond to Eu^2+^ 4f–5d transitions. These align well with the diffuse reflectance data: Figure S10 shows that the undoped strontium aluminate hosts all have a fundamental absorption edge near ~230 nm, but the Eu-doped samples exhibit an absorption tail extending further into the UV as *x* increases (reaching ~410 nm for *x* = 4). This red-shift of the absorption edge/bandgap with higher Al content (*x*) is also reflected in the Tauc plot analysis of optical bandgaps (Figure S11) [42]: the bandgap (E_g_) narrows from ~4.86 eV at *x* = 1/2 to ~2.70 eV at *x* = 4. The incorporation of more Al likely introduces defect levels or alters the lattice enough to allow Eu^2+^ 5d states to be stabilized at lower energies, hence absorbing longer wavelengths. In summary, the excitation spectra and emission behavior highlight a key advantage of the Eu-doped strontium aluminate system: by selecting the excitation wavelength, one can further tune the output color for certain compositions. This is especially pronounced for the *x* ~1–3/2 compositions where both Eu^2+^ and Eu^3+^ are present. Such excitation-dependent color switching is valuable for applications like dynamic displays or sensors that respond differently to two excitation sources.

**Figure 6:**
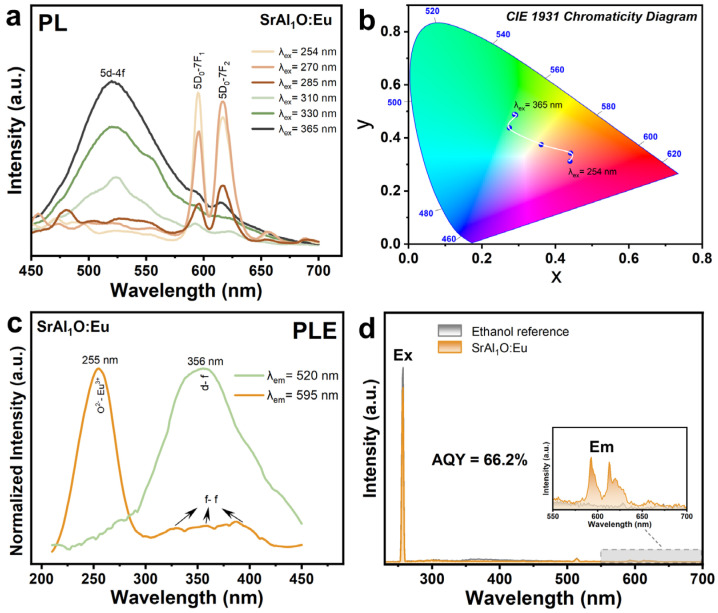
The excitation-dependent luminescence of Eu-doped strontium aluminate phosphor (*x* = 1). (**a**) PL spectra for the *x* = 1 phosphor under different excitations from 254 to 365 nm, undergoing a transition from red (dominant Eu^3+^ emission) to orange, yellow and, finally, green (dominant Eu^2+^ emission). (**b**) CIE chromaticity diagram corresponding to the spectra in (**a**), with the coordinates moving from the red region to green as the excitation wavelength increases. (**c**) PLE spectra of the *x* = 1 phosphor for 520 and 595 nm, exhibiting excitation peaks at ~365 nm (Eu^2+^) and ~255 nm (Eu^3+^ charge-transfer), respectively. (**d**) Measurement of the absolute quantum yield (AQY) for the *x* = 1 phosphor, which gives the highest value of 66.2%.

To illustrate this, we focus on the Eu-doped strontium aluminate (*x* = 1) phosphor that contains a mixed phase (Sr_3_Al_2_O_6_ + SrAl_2_O_4_) and mixed-valence Eu. Figure 6a shows its emission spectra under a series of excitation wavelengths from 254 nm to 365 nm. At 254, 270 and 285 nm (short-UV), the spectrum is dominated by Eu^3+^ emissions at 593 and 617 nm (red–orange). At longer excitations (310, 330 and 365 nm), a broad Eu^2+^ emission band (~520 nm) emerges and eventually dominates, while the Eu^3+^ peaks diminish. The gradual shift in color is visualized in the CIE plot in Figure 6b, where the coordinate moves from the red region (under 254 nm) to green (under 365 nm). The corresponding CIE coordinates are listed in Table S5. This behavior is due to the differential excitation of Eu^3+^ vs. Eu^2+^, as discussed above. It also provides insight into Eu^3+^ site symmetry: for example, the relative intensity of the Eu^3+^ 593 nm vs. 617 nm lines changes with excitation wavelength. We observe that 254 nm excitation yields a higher 593/617 ratio than 270 nm excitation, implying that at slightly different excitation energies, Eu^3+^ ions at different lattice sites (with slightly different environments) are preferentially excited [41]. This subtle effect reflects the presence of multiple distinct Eu^3+^ sites (Site A with inversion symmetry; Site B without, as depicted earlier in Figure 2d). In essence, the Eu-doped strontium aluminate (*x* = 1) phosphor acts as a responsive multicolor phosphor, changing its emission color depending on the excitation source.

The excitation spectra corresponding to different emission bands further clarify the valence-selective excitation behavior of Eu ions in the *x* = 1 phosphor (Figure 6c). For the 520 nm emission, the excitation profile exhibits a broad band centered at ~365 nm, which is characteristic of the allowed 4f^6^5d^1^ → 4f^7^ transition of Eu^2+^. In contrast, when monitoring the 595 nm emission, the excitation spectrum contains a strong band centered at ~255 nm, together with several weaker features in the 300–400 nm region. The ~255 nm band is assigned to the charge-transfer band (CTB) from O^2−^ 2p orbitals to the partially filled 4f^6^ levels of Eu^3+^, whereas the weaker bands originate from parity-forbidden 4f-4f transitions of Eu^3+^ [35,41]. At high-energy excitation (λ < 285 nm), photons readily excite the CTB of Eu^3+^, enabling efficient electron transfer from O^2−^ to Eu^3+^ and thus promoting Eu^3+^ emission. In the long-wavelength UV region (310–365 nm), the photon energy is insufficient to activate the CTB or higher-lying 4f-4f transitions of Eu^3+^, but it aligns well with the 4f^6^5d^1^ excitation energy of Eu^2+^, thereby effectively triggering the broad-band Eu^2+^ emission. Because the excitation features of Eu^2+^ and Eu^3+^ exhibit negligible overlap across 210–450 nm, selective excitation becomes feasible, enabling continuous modulation of emission color by tuning the excitation wavelength.

Finally, among our samples, the Eu-doped strontium aluminate phosphor with *x* = 1 stands out for its exceptionally high quantum efficiency. Under 365 nm excitation, it emits bright green light with an AQY of 66.2% (Figure 6d), which is the highest value measured in this work. This AQY is substantially higher than those of the other compositions (Figures S2 and S12), which range from only a few percent up to ~46% (e.g., SrAl_2_O_4_:Eu). To contextualize this performance, we compared our AQY values with the reported Eu-activated phosphors (both Eu^2+^ and Eu^3+^), as summarized in Table S6. Notably, both SrAl_2_O_4_:2 at%Eu^2+^ (green emission, AQY 46.5%) and the *x* = 1 phosphor (high-efficiency emission, AQY 66.2%) rank among the top-performing Eu-doped phosphors in terms of quantum yield. By comparison, many Eu^2+^-activated silicate or sulfide phosphors typically exhibit AQYs in the ~10–40% range, while Eu^3+^-activated materials often remain below 50% [43,44,45,46]. Previous research has emphasized that the selection of the host matrix is crucial for achieving high quantum yield, as host materials with low phonon energy and high structural rigidity can effectively minimize non-radiative energy loss [47,48,49]. The outstanding efficiency of the *x* = 1 phosphor can be attributed to a favorable combination of activator behavior and host characteristics: the host lattice provides a rigid, low-vibrational environment that suppresses non-radiative relaxation, while the Eu-related emission pathways are well supported by the local coordination environment, enabling efficient radiative recombination. On the one hand, the mixed-phase (Sr_3_Al_2_O_6_/SrAl_2_O_4_) strontium aluminate host is known to possess a rigid lattice with low phonon energy [50,51]. Low phonon energy is critical for suppressing non-radiative relaxation—high-frequency lattice vibrations, which dissipate excitation energy as heat instead of light, are effectively minimized by this structural feature. Similar to the coordination-induced structural rigidity strategy reported to enhance quantum efficiency in phosphorescent materials, the rigid lattice of our mixed-phase host inhibits lattice vibrations and reduces non-radiative decay pathways [51]. On the other hand, the local coordination environment of Eu ions in the mixed-phase host provides moderate crystal field splitting of the Eu 4f and 5d orbitals. This moderate splitting optimizes the energy level matching between the excited and ground states of both Eu^2+^ and Eu^3+^, thereby promoting efficient radiative recombination [52]. Specifically, the crystal field strength reduces the probability of non-radiative transitions (e.g., cross-relaxation between Eu ions) and enhances the radiative transition probabilities of Eu^2+^ (4f^6^5d^1^ → 4f^7^) and Eu^3+^ (^5^D_0_ → ^7^F_j_), which can significantly improve quantum yield. This high AQY makes the *x* = 1 Eu-doped strontium aluminate particularly promising not only for display and optical tagging, but also for luminescence-based sensing applications, as discussed in the following section.

### 3.4. Thermal Quenching and Luminescence Thermometry

For luminescent materials intended for practical use (e.g., LEDs, optical tags, or sensors), thermal stability of emission is a key performance parameter. We therefore examined the temperature-dependent PL behavior of our phosphors to assess how emission intensity and luminescence performance are preserved at elevated temperatures. As shown in Figure S13, the emission spectra of three representative samples (Sr_3_Al_2_O_6_:Eu, SrAl_2_O_4_:Eu and Sr_4_Al_14_O_25_:Eu) were recorded from 300 K up to 570 K under 254 nm excitation. With increasing temperature, all three samples exhibit a gradual decrease in emission intensity due to thermal quenching, but the extent of quenching differs markedly among the phases. At 420 K, Sr_3_Al_2_O_6_:Eu retained ~56% of its room-temperature intensity, whereas SrAl_2_O_4_:Eu dropped to ~40% and Sr_4_Al_14_O_25_:Eu to ~20%. These results indicate that the Al-rich phases (higher *x*) undergo substantially stronger thermal quenching and thus exhibit poorer emission stability at high temperature. At 570 K, Sr_3_Al_2_O_6_:Eu still maintained ~17% of its initial intensity, while SrAl_2_O_4_:Eu and Sr_4_Al_14_O_25_:Eu were nearly quenched. These results indicate that the Al-rich phases (higher *x*) undergo substantially stronger thermal quenching and thus exhibit poorer emission stability at high temperature. The likely reason is that Al-rich lattices contain higher concentrations of defects (e.g., oxygen vacancies or disorder), which act as quenching centers. With increasing temperature, excited-state energy can migrate via these defect states and be non-radiatively deactivated, causing a rapid drop in luminescence [53]. In contrast, the Sr_3_Al_2_O_6_ host (lower Al content) appears to have fewer such quenching pathways, resulting in comparatively greater thermal robustness.

To further quantify the quenching behavior, we extracted the thermal activation energy (E_a_) by fitting the integrated PL intensity as a function of temperature using an Arrhenius-type expression: *I*(*T*) = *I*_0_/[1 + *C*exp (−E_a_/*kT*)] [54]. The fitted results (insets of Figure S13) yield E_a_ ≈ 0.22 eV for Sr_3_Al_2_O_6_:Eu, 0.37 eV for SrAl_2_O_4_:Eu and 0.40 eV for Sr_4_Al_14_O_25_:Eu. Although the Al-rich phases exhibit larger E_a_ values, their poorer thermal stability is not necessarily contradictory. Instead, it likely reflects different dominant quenching pathways: defect-mediated quenching in SrAl_2_O_4_ and Sr_4_Al_14_O_25_ may involve higher barriers but also a much denser network of defect/trap states, enabling efficient non-radiative deactivation at relatively lower temperatures despite the larger apparent E_a_ [55]. Overall, the key outcome is clear: emission retention decreases systematically with increasing Al content, highlighting the strong phase dependence of thermal quenching in this system.

**Figure 7:**
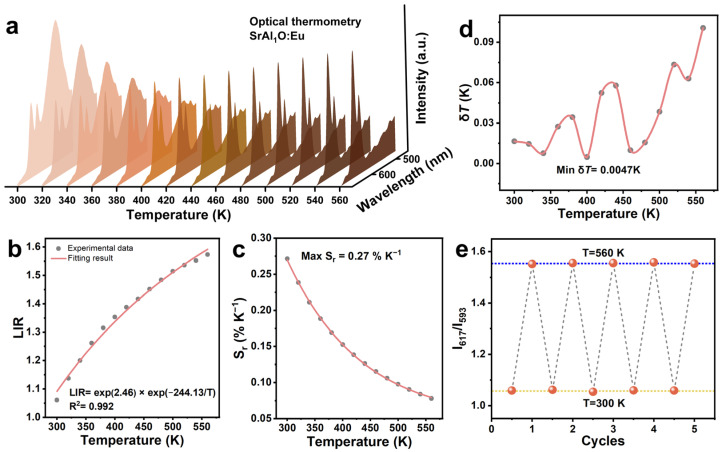
Optical thermometry based on the Eu^3+^ emission intensity ratio in the Eu-doped strontium aluminate phosphor (*x* = 1). (**a**) Temperature-dependent emission spectra of the *x* = 1 phosphor recorded under λ_ex_ = 300 nm from 300 to 560 K. (**b**) Experimentally measured *LIR* (*I*_617_/*I*_593_) values as a function of temperature, together with an exponential fit. (**c**) Relative sensitivity (*Sᵣ*) of the *x* = 1 phosphor over 300–560 K. (**d**) Temperature uncertainty (*δT*) as a function of temperature. (**e**) Cycling stability of the thermometric parameter: *I*_617_/*I*_593_ measured at 300 K and 560 K over five consecutive heating–cooling cycles, demonstrating durability under repeated temperature cycling.

Interestingly, the Eu-doped strontium aluminate phosphor with *x* = 1 exhibits an anomalous temperature response: rather than showing purely monotonic thermal quenching, its emission changes in character as the temperature increases. As shown in Figure 7a, the emission spectra recorded from 300 K to 560 K under 300 nm excitation (to simultaneously excite both Eu^3+^ and Eu^2+^ contributions) reveal two opposing trends. With increasing temperature, the Eu^2+^ broad-band emission centered at ~511 nm decreases to roughly 20% of its initial intensity, consistent with conventional thermal quenching. In contrast, the Eu^3+^ line emissions at ~593 nm and ~617 nm become stronger as temperature rises: at 560 K, the 593 nm line increases to approximately 1.1×, and the 617 nm line to about 1.6×, relative to their intensities at 300 K. This counterintuitive enhancement in Eu^3+^ emission is plausibly associated with thermally assisted energy transfer and trap-mediated feeding processes. At elevated temperatures, part of the excited-state energy of Eu^2+^ (4f^6^5d^1^) may be more efficiently transferred to nearby Eu^3+^ centers (or Eu^3+^-related trap states) prior to Eu^2+^ radiative relaxation, thereby populating the Eu^3+ 5^D_0_ level and enhancing Eu^3+^ emission. In addition, higher thermal energy can promote the release of trapped charge carriers that preferentially recombine through Eu^3+^-associated pathways, further amplifying the Eu^3+^ line intensities [56,57]. As a consequence, the overall emission color of the *x* = 1 phosphor shifts toward a stronger Eu^3+^ (red) contribution at higher temperatures.

This temperature-dependent redistribution of emission provides a useful basis for ratiometric optical thermometry using a luminescence intensity ratio (*LIR*). We define the *LIR* as *LIR* = *I*_617_/*I*_593_, where I_617_ and I_593_ are the peak intensities of the Eu^3+^ emissions at 617 nm (red) and 593 nm (orange), respectively. As shown in Figure 7b, the *LIR* increases from ~1.06 at 300 K to ~1.57 at 560 K, indicating a strong and monotonic temperature dependence. The experimental data can be empirically fitted by *LIR* = *exp*(2.46) × *exp*(−244.13/*T*) (T in K), which can be linearized as ln(*I*_617_/*I*_593_) = 2.46 − 244.13(1/*T*). Consistent with this, Figure S14 shows a good linear relationship between ln(*LIR*) and 1/*T* over the 300–560 K range. Although the fitted slope (−244.13) can be converted into an apparent activation energy (~0.0209 eV), it is most appropriately interpreted here as a phenomenological calibration parameter rather than a direct representation of a single elementary process. Importantly, this calibration enables the temperature to be determined directly from a single emission spectrum via the measured *LIR*, which is the fundamental operating principle of luminescence-based optical thermometry.

Two key performance metrics for luminescence-based optical thermometers are the relative sensitivity (*S_r_*) and the temperature uncertainty (*δT*). The relative sensitivity quantifies the fractional change in the luminescence intensity ratio per unit temperature and is defined as *S_r_* = 1/*LIR*·d(*LIR*)/d*T*. A larger *S_r_* indicates a stronger temperature response and therefore better sensing capability. The temperature uncertainty *δT* reflects the smallest resolvable temperature variation and can be estimated by *δT* = (*δ*(*LIR*)/*LIR*)/*S_r_*, where *δ*(*LIR*)/*LIR* represents the relative measurement error of the *LIR* (typically obtained from repeated measurements) [58,59,60]. We evaluated these parameters for the Eu-doped strontium aluminate phosphor (*x* = 1). As shown in Figure 7c, *Sᵣ* exhibits a clear temperature dependence, increasing as temperature decreases, consistent with the stronger slope d(*LIR*)/d*T* at lower temperature. The maximum sensitivity reaches approximately 0.27% K^−1^ at 300 K and remains on the order of 0.1–0.2% K^−1^ throughout the 300–560 K range. These values are comparable to—and, in some cases, competitive with—many reported lanthanide-based thermometers (Table S7), which demonstrates the great development potential of Eu-doped strontium aluminate phosphors in optical temperature sensing applications.

In parallel, the temperature uncertainty is exceptionally small. Using repeated measurements to determine *δ*(*LIR*), we find that *δT* remains below 0.1 K across the full 300–560 K interval and reaches a minimum of approximately 0.0047 K near 400 K (Figure 7d). This very low uncertainty reflects the excellent repeatability of the Eu^3+^ emission intensities, in particular, the high reproducibility of the *I*_617_/*I*_593_ ratio with minimal fluctuation across measurements. We further assessed the cycling stability of the thermometer response. The sample was repeatedly heated and cooled between 300 K and 560 K for five cycles, and the *LIR* was recorded at both temperature endpoints during each cycle. As shown in Figure 7e, the *I*_617_/*I*_593_ ratio returns to essentially the same values in every cycle, with no detectable drift or degradation. Consistent with this, the post-cycling XRD pattern (Figure S15) shows no new phases and no appreciable peak shifts, confirming that the host lattice remains structurally intact and does not undergo detectable thermal decomposition. Overall, these results demonstrate that the *x* = 1 phosphor functions as an excellent photoluminescent temperature sensor in the 300–560 K range, combining high sensitivity, very low temperature uncertainty, and excellent reversibility and thermal robustness, all of which are essential for practical sensing applications.

### 3.5. Photothermal Conversion Under NIR Irradiation

Beyond their photoluminescent behavior, the Eu-doped strontium aluminate phosphors investigated here can also function as photothermal conversion materials. The Eu-related electronic states (involving 4f and 5d levels) enable light absorption extending into the NIR region. Upon NIR excitation, the absorbed energy can be dissipated through non-radiative relaxation pathways, leading to an increase in lattice vibrations and thus heat generation. Efficient NIR-to-heat conversion is attractive for a range of applications, including thermal imaging, photothermal therapy (subject to biocompatibility considerations), and solar/photothermal energy harvesting [61,62]. Accordingly, we evaluated the photothermal response of representative phosphors under 808 nm and 980 nm laser irradiation, as well as under simulated sunlight, following the procedures described in the Experimental Section 2.

**Figure 8:**
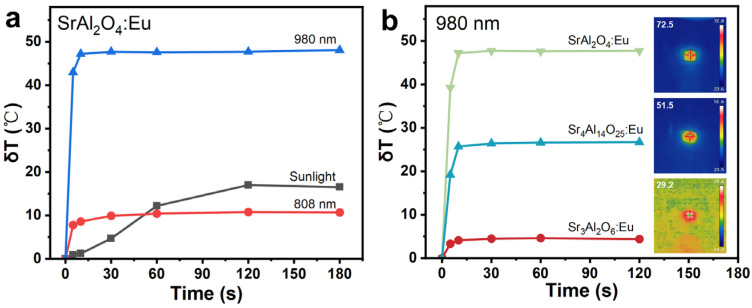
Photothermal response of Eu-doped strontium aluminate phosphors under NIR laser and simulated sunlight irradiation. (**a**) Time-dependent temperature evolution of SrAl_2_O_4_:Eu under 808 nm and 980 nm laser- and Xe-lamp-simulated light, with all light sources operated at the same power density (1 W·cm^−2^). (**b**) Representative infrared thermal images recorded after 120 s of irradiation, visualizing the light-to-heat conversion performance of different Eu-doped strontium aluminate compositions under identical conditions.

Among the compositions studied, the SrAl_2_O_4_:Eu phosphor (*x* = 2) exhibits the most pronounced photothermal response. Figure 8a shows the net temperature rise (Δ*T*) of SrAl_2_O_4_:Eu as a function of irradiation time under three excitation sources, each operated at the same power density (1 W·cm^−2^): an 808 nm laser, a 980 nm laser and simulated solar illumination. Under 980 nm NIR irradiation, the sample heats rapidly and reaches a steady-state temperature increase of ΔT ≈ 48.0 °C, rising from ambient (~24 °C) to approximately 72 °C within ~10 s. By comparison, irradiation at 808 nm produces only a modest increase of ~10.7 °C within ~30 s, while simulated sunlight yields a moderate rise of ~16.5 °C within ~120 s under the same power density. These results indicate that SrAl_2_O_4_:Eu couples much more effectively to 980 nm photons, enabling efficient conversion of absorbed optical energy into heat, whereas absorption at 808 nm is significantly weaker. The intermediate response under simulated sunlight is expected, given its broad spectral distribution across the visible and NIR regions. The strong photothermal response under 980 nm excitation is particularly attractive from an application standpoint, since 980 nm is a widely used NIR wavelength with good penetration through many media (e.g., a biological window), enabling practical deployment in remotely triggered heating and related photothermal technologies.

We next compared the photothermal responses of different Eu-doped strontium aluminate compositions under 980 nm excitation since this wavelength produced the strongest heating effect. Figure 8b shows the time-dependent temperature profiles of Sr_3_Al_2_O_6_:Eu (*x* = 1/2), SrAl_2_O_4_:Eu (*x* = 2) and Sr_4_Al_14_O_25_:Eu (*x* = 4) under a 980 nm laser (1 W·cm^−2^). A clear compositional dependence is observed. SrAl_2_O_4_:Eu reaches the highest steady-state temperature of approximately 72.5 °C (Δ*T* ≈ 47.7 °C) within 10 s, whereas Sr_3_Al_2_O_6_:Eu shows only a small temperature rise to ~29.2 °C (Δ*T* ≈ 4.4 °C), and Sr_4_Al_14_O_25_:Eu reaches ~51.5 °C (Δ*T* ≈ 26.7 °C). The infrared thermal images (inset of Figure 8b) further corroborate this trend, visually confirming that SrAl_2_O_4_:Eu heats much more strongly than the other phases under identical irradiation conditions. Collectively, these results identify the *x* = 2 composition as the most efficient photothermal converter among the three representative phases.

The superior photothermal performance of SrAl_2_O_4_:Eu likely arises from a combination of optical absorption and non-radiative relaxation characteristics. Its moderate effective bandgap (~3.0 eV, Figure S11) may support stronger NIR absorption through sub-bandgap tail states, Eu-related absorption features, and/or defect-assisted pathways while also providing efficient non-radiative channels for converting absorbed energy into lattice heat. By contrast, the Al-poor Sr_3_Al_2_O_6_:Eu has a bandgap that is too wide (~4.86 eV) to absorb NIR efficiently, resulting in negligible heating. Although the Al-rich Sr_4_Al_14_O_25_:Eu has a narrower bandgap (~2.7 eV), its excitation pathways and local environment may favor radiative emission more strongly, reducing the fraction of absorbed energy converted to heat. In this context, SrAl_2_O_4_:Eu appears to offer an optimal balance—sufficient NIR coupling combined with effective phonon-mediated dissipation—leading to the highest temperature rise. From an application perspective, the rapid and substantial heating achieved by SrAl_2_O_4_:Eu under 980 nm excitation shows strong potential for NIR-triggered photothermal coatings and devices. Moreover, because SrAl_2_O_4_:Eu can also exhibit luminescent responses under NIR excitation, this material system may enable integrated photothermal and photoluminescent readouts, offering a route toward multifunctional sensing and smart thermal-response platforms.

## 4. Conclusions

In summary, a series of Eu-doped strontium aluminate phosphors (*x* = 1/2–4) with continuous full-visible photoluminescence tunability and multiple functionalities was developed. Adjusting the Al/Sr ratio (*x*) regulated the host crystal structure (Sr_3_Al_2_O_6_ → SrAl_2_O_4_ → Sr_4_Al_14_O_25_ as *x* increases) and Eu valence state/site occupancy: low-*x* compositions emit Eu^3+^-centered red–orange light, while Al-rich ones show Eu^2+^-centered green–blue emission, enabling full-color control in one material. The intermediate composition (*x* = 1, mixed phases, coexisting Eu^2+^/Eu^3+^) exhibits excitation-wavelength-dependent luminescence, switching from red (high-energy excitation) to green (long-wavelength UV region excitation). Based on the Eu^3+^ emission intensity ratio, a ratiometric optical thermometer was constructed, achieving a high relative sensitivity of approximately 0.27% K^−1^ and an ultralow temperature uncertainty of ~0.005 K at 300–560 K (excellent reversibility/stability). Practical applications include anti-counterfeiting/information display luminescent inks and superior NIR photothermal conversion (72.8 °C within 10 s at 1 W·cm^−2^) of SrAl_2_O_4_:Eu. This work provides a composition-driven strategy for multifunctional phosphors, meeting diverse application demands and offering guidance for advanced composite phosphor development via compositional modulation.

## Data Availability

The original contributions presented in this study are included in this article/Supplementary Material. Further inquiries can be directed to the corresponding author.

## Supplementary Materials

The following supporting information can be downloaded at: https://www.mdpi.com/article/10.3390/ma19112351/s1.
