# Supplementary material for: Composition-Modulated Strontium Aluminate Phosphors with Continuously Tunable Visible Emission for Advanced Display, Thermometry and Photothermal Conversion
Source: Materials (Basel). 2026 Jun 2;19(11):2351. doi: 10.3390/ma19112351 (PMC13258628; doi:10.3390/ma19112351)
Supplement: Supplementary file 1 [file materials-19-02351-s001.zip › materials-4293774-supplementary.pdf]

Supplementary Materials

Composition-modulated strontium aluminate phosphors with continuously tunable visible emission for advanced display, thermometry and photothermal conversion

Jingwen Yang[1] and Guijian Guan[1,2]*

[1]Institute of Molecular Plus, Tianjin University, Tianjin, 300072, P. R. China; yjw0326_@tju.edu.cn.

[2]State Key Laboratory of Advanced Papermaking and Paper-based Materials, South China University of Technology, Guangzhou 510640, P. R. China; guijianguan@tju.edu.cn.

Correspondence: guijianguan@tju.edu.cn.

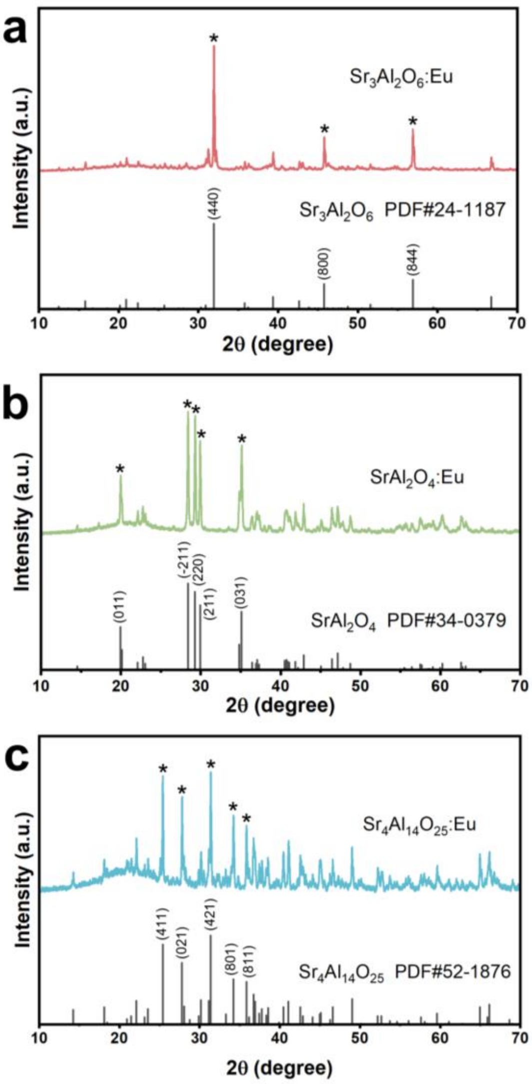

Figure S1. XRD patterns confirming phase control via the Al/Sr ratio. (a) $Sr_3Al_2O_6$:Eu (synthesized from precursors at Al/Sr = 1/2). (b) $SrAl_2O_4$:Eu (Al/Sr = 2). (c) $Sr_4Al_{14}O_{25}$:Eu (Al/Sr = 4). All samples are doped with 2 at% Eu; tick marks indicate reference peak positions for the respective phases.

Table S1. Ionic radii of relevant ions in Eu-doped strontium aluminate phosphors [1]. The close match between the radius of Eu^{2+} and Sr^{2+} explains Eu's preference for Sr sites in the lattice.

**Ion**	Sr^{2+}	Al^{3+}	O^{2-}	Eu^{2+}	Eu^{3+}
**Ionic radius (Å)**	1.18	0.53	1.40	1.20	0.95

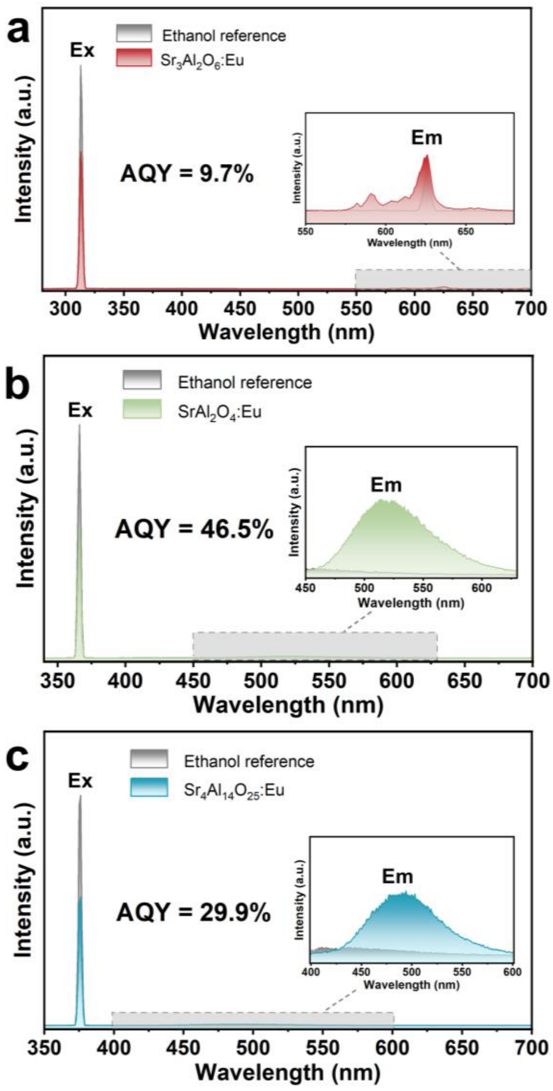

Figure S2. Absolute quantum yield (AQY) measurements for Eu-doped strontium aluminate phosphors (using ethanol as a reference). (a) Emission and excitation spectra of $Sr_3Al_2O_6$:Eu and ethanol, yielding AQY ~ 9.7%. (b) Spectra for $SrAl_2O_4$:Eu and ethanol, yielding AQY ~ 46.5%. (c) Spectra for $Sr_4Al_{14}O_{25}$:Eu and ethanol, yielding AQY ~ 29.9%. Here, AQY is defined as the ratio of emitted photons to absorbed photons, calculated from integrated emission and excitation areas after subtracting the ethanol background.

Table S2. Excitation (λ_{ex}) and emission (λ_{em}) wavelengths used for AQY measurements of Eu-doped strontium aluminate samples (x = 1/2, 1, 3/2, 2, 3, 4).

***x* = Al/Sr for samples**	**λ_{ex} (nm)**	**λ_{em} (nm)**
1/2	312	617
1	255	595

3/2	355	520
2	368	520
3	374	492
4	374	490

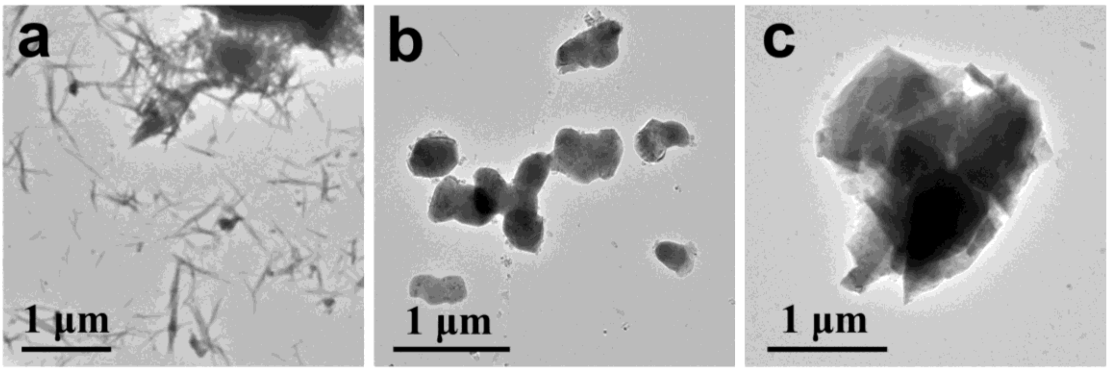

Figure S3. Morphologies of Eu-doped strontium aluminate phosphors. TEM images indicate nano-needle morphology for (a) $Sr_3Al_2O_6$:Eu, granular particles for (b) $SrAl_2O_4$:Eu and block-like grains for (c) $Sr_4Al_{14}O_{25}$:Eu.

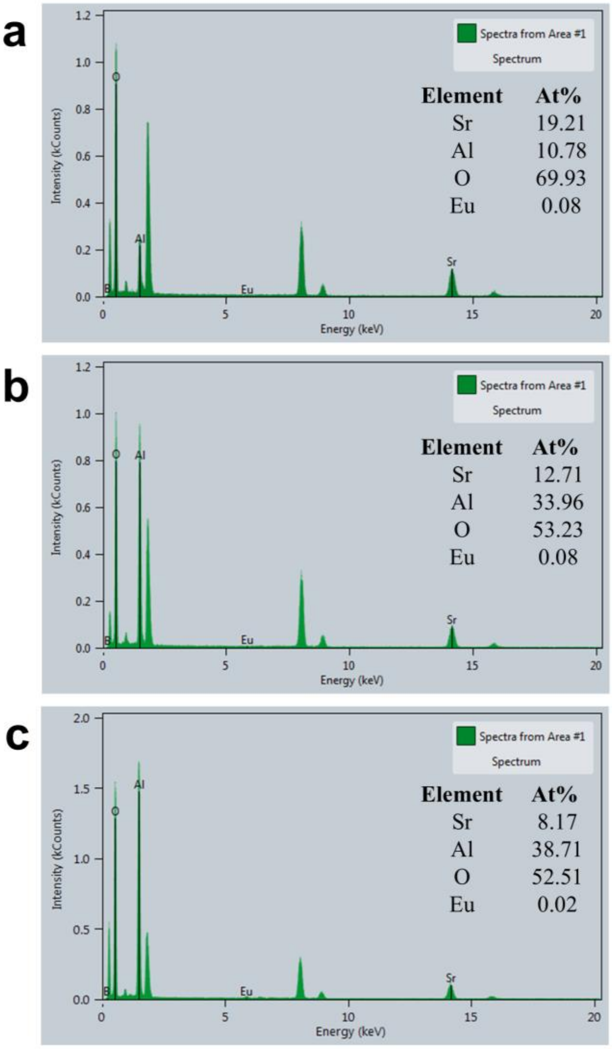

Figure S4. EDS spectra and elemental analysis of selected Eu-doped strontium aluminate phosphors: (a) $Sr_3Al_2O_6$:Eu, (b) $SrAl_2O_4$:Eu, and (c) $Sr_4Al_{14}O_{25}$:Eu. The measured Al/Sr molar ratios are in close agreement with the expected stoichiometry for each sample, confirming successful composition control.

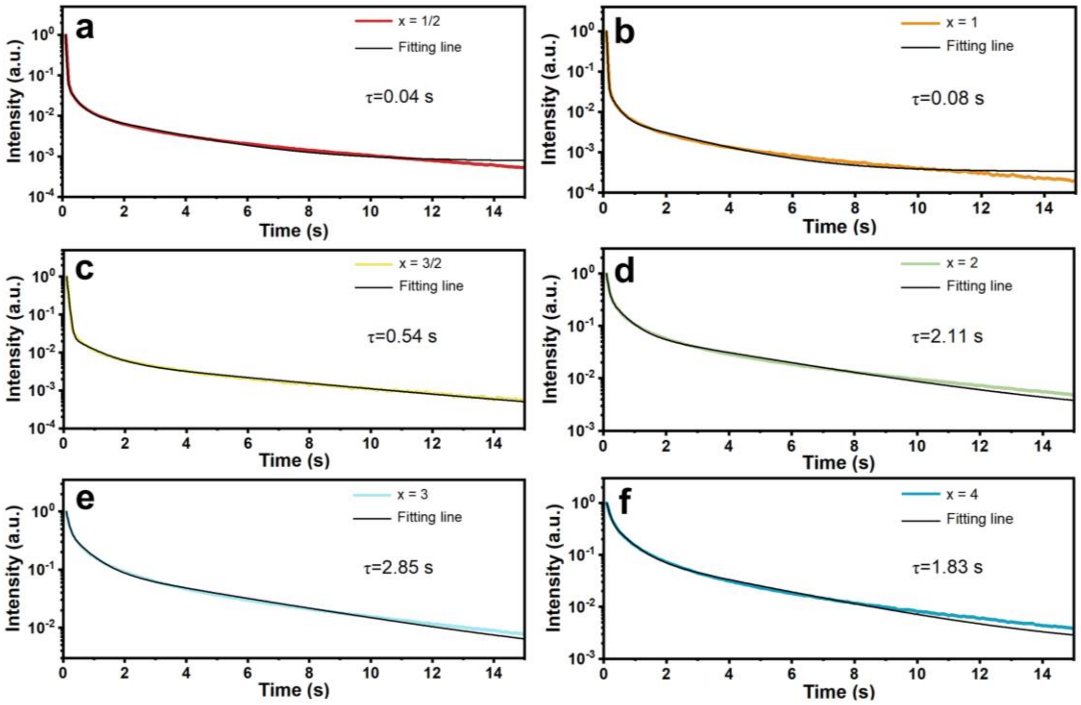

Figure S5. Photoluminescence decay curves (under 254 nm excitation) for Eu-doped strontium aluminate phosphors (x = 1/2, 1, 3/2, 2, 3, 4). Black lines are tri-exponential fits. The extracted average decay lifetimes (τ) are 0.04 s, 0.08 s, 0.54 s, 2.11 s, 2.85 s, and 1.83 s for x = 1/2, 1, 3/2, 2, 3, and 4, respectively. A higher Al content generally leads to a longer phosphorescence lifetime, likely due to trap states in the Al-rich phases.

Table S3. Tri-exponential fitting parameters (amplitudes a_i and time constants τ_i) for the photoluminescence decay curves of the Eu-doped strontium aluminate phosphors (x = 1/2-4) and the calculated average lifetime τ_{avg} for each sample. Here, τ_{avg} is computed using $\tau_{avg} = (a_1\tau_1^2 + a_2\tau_2^2 + a_3\tau_3^2)/(a_1\tau_1 + a_2\tau_2 + a_3\tau_3)$.

***x* = Al/Sr for samples**	$\mathbf{a_1}$	$\boldsymbol{\tau_1}$	$\mathbf{a_2}$	$\boldsymbol{\tau_2}$	$\mathbf{a_3}$	$\boldsymbol{\tau_3}$	$\boldsymbol{\tau_{avg}}$ **(s)**
1/2	93.486	0.0219	0.0461	0.2481	0.0071	2.0326	0.04
1	0.0689	0.2826	0.0127	2.4696	58.6317	0.0242	0.08
3/2	6.9089	0.0510	0.0266	0.7328	0.0061	5.5612	0.54
2	2.8766	0.0662	0.3551	0.4950	0.0749	4.2375	2.11
3	1.6454	0.0904	0.4111	0.6232	0.1045	4.8043	2.85
4	1.5406	0.0911	0.4636	0.5588	0.0987	3.4903	1.83

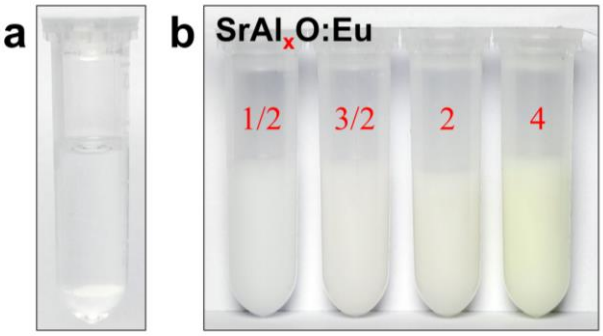

Figure S6. Preparation of phosphor-based epoxy inks. (a) Photograph of the transparent epoxy resin carrier fluid obtained after the epoxy–amine reaction (resin + APTES). (b) Photographs of epoxy-based luminescent inks mixed with different Eu-doped strontium aluminate phosphors (x = 1/2, 3/2, 2, 4), showing uniformly dispersed colors.

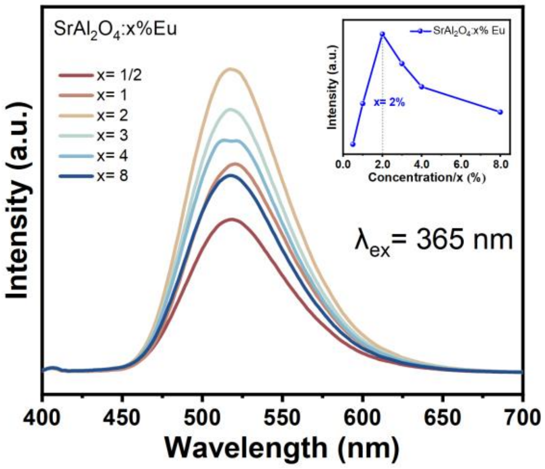

Figure S7. PL spectra of $SrAl_2O_4$:x%Eu with varying Eu doping concentrations (1/2 ≤ x ≤ 8) under 254 nm excitation (inset: emission intensity at 520 nm vs Eu concentration). The emission peak position (~520 nm) remains constant, but intensity increases up to 2 at% Eu and then diminishes due to concentration quenching.

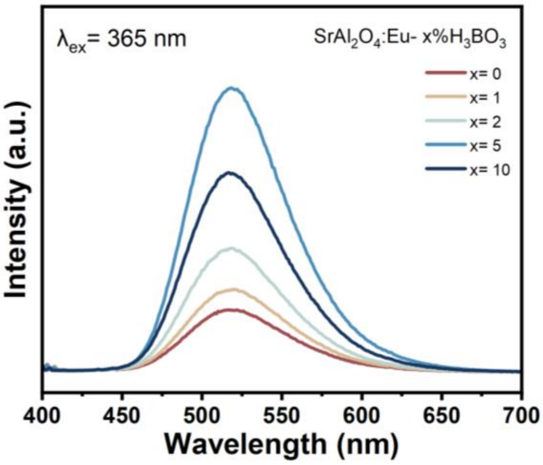

Figure S8. PL spectra of $SrAl_2O_4$:Eu synthesized with varying H_3BO_3 flux amounts (0, 1, 2, 5, 10 at%) at 254 nm excitation. The emission peak position (~520 nm) does not shift with flux, but intensity is maximized at 5 at% H_3BO_3. Higher flux (10 at%) causes larger particle growth and reduces luminescence.

Table S4. CIE 1931 chromaticity coordinates (x,y) of the emission colors of Eu-doped strontium aluminate phosphors under 254 nm excitation. The table shows the color progression from red to blue obtained as the Al/Sr ratio (*x*) increases from 1/2 to 4.

***x* = Al/Sr for samples**	**CIE (x, y)**	**Color**
1/2	(0.1694, 0.3413)	Red
1	(0.1407, 0.3865)	Orange
3/2	(0.2453, 0.5307)	Yellow
2	(0.3507, 0.3830)	Green
3	(0.4090, 0.3089)	Cyan
4	(0.3306, 0.2562)	Blue

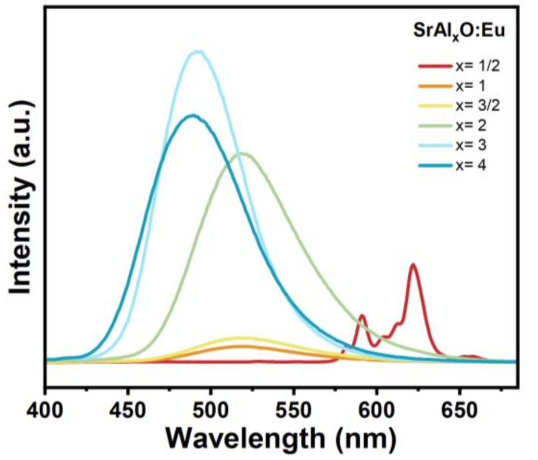

Figure S9. PL spectra of Eu-doped strontium aluminate phosphors under their optimal excitation wavelengths (optimal λ_{ex}: 312 nm for x = 1/2; 350 nm for x = 1; 355 nm for x = 3/2; 365 nm for x = 2; 374 nm for x = 3 and x = 4). Each spectrum was measured at the wavelength that yields maximum intensity for that sample.

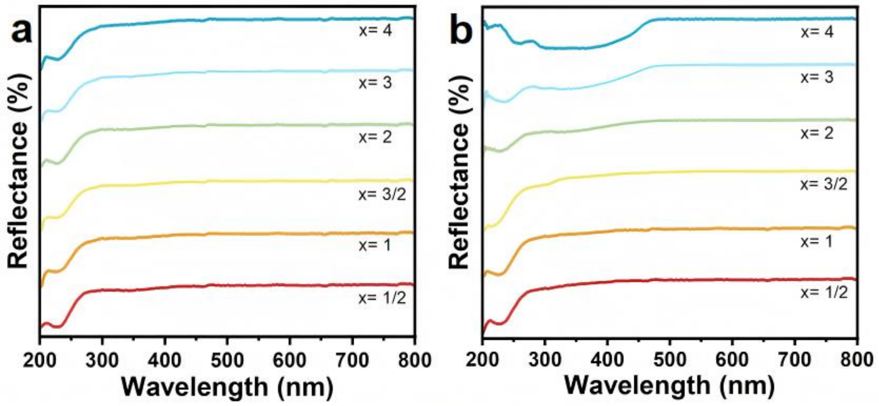

Figure S10. Diffuse reflectance spectra analysis. (a) Diffuse reflectance spectra of undoped strontium aluminate hosts (x = 1/2-4): all show a fundamental absorption edge around ~230 nm (~5.4 eV). (b) Diffuse reflectance spectra of Eu-doped strontium aluminate phosphors: as x increases, an absorption tail extends into the UV–visible region (red-shifting the effective band edge up to ~411 nm for x = 4). Eu doping introduces 4f–5d absorption bands and defect levels that lower the effective optical bandgap.

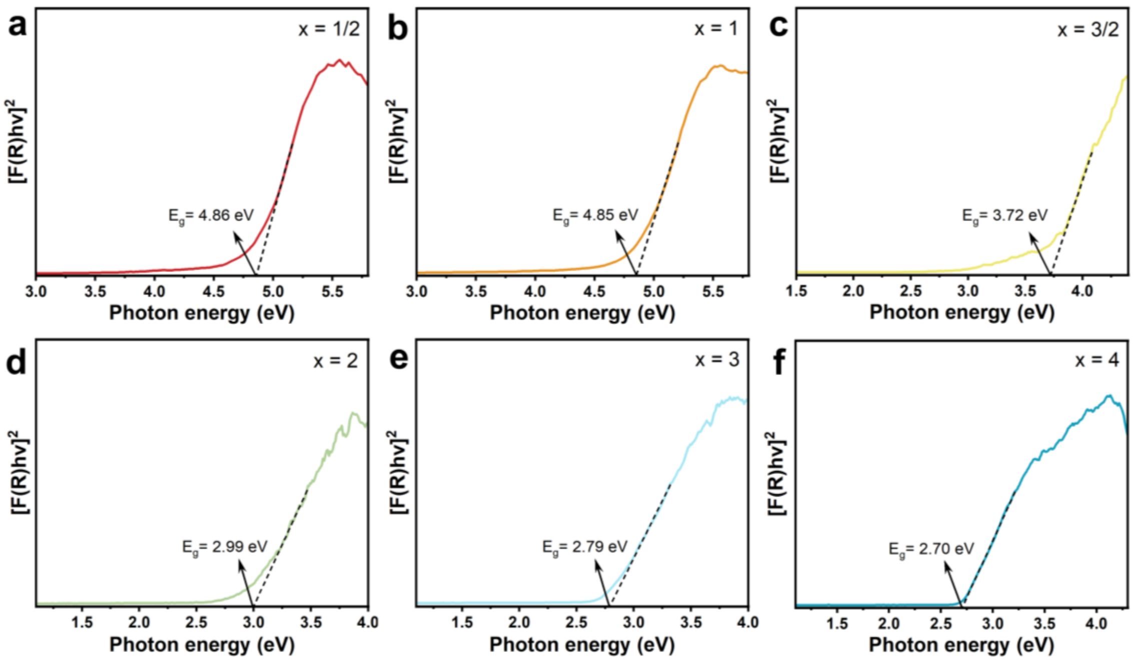

Figure S11. Tauc plot for determining the optical bandgaps of Eu-doped strontium aluminate phosphors. Plots of $[F(R)hv]^2$ vs hv (where F(R) is the Kubelka–Munk function) for x = 1/2, 1, 3/2, 2, 3, and 4 yield E_g values of 4.86, 4.85, 3.72, 2.99, 2.79, and 2.70 eV, respectively. A higher Al content (x) leads to a smaller bandgap, consistent with the trend observed in diffuse reflectance spectra.

Table S5. CIE chromaticity coordinates of Eu-doped strontium aluminate (x = 1) emission under different excitation wavelengths (corresponding to **Figure 5**b). These coordinates illustrate the excitation-dependent color shift from red to green.

λ_{ex} (nm)	CIE (x, y)	Color
254	(0.2884, 0.4889)	Red
270	(0.2916, 0.4863)	Orange
285	(0.2748, 0.4380)	Yellow
310	(0.3613, 0.3746)	Green
330	(0.4415, 0.3426)	Green
365	(0.4397, 0.3118)	Green

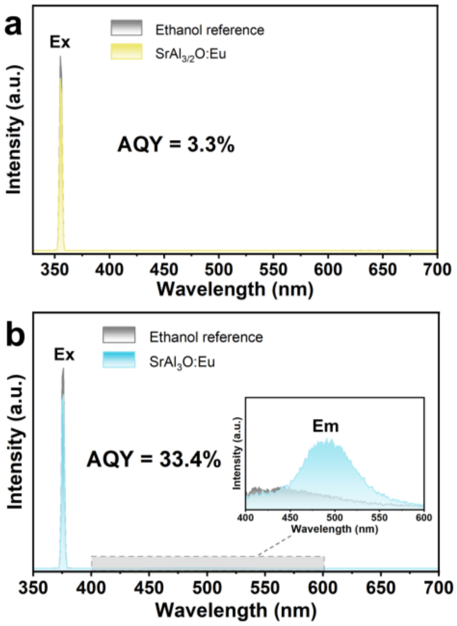

Figure S12. Additional AQY measurements for intermediate compositions. (a) PL spectra of Eu-doped strontium aluminate (x = 3/2) and ethanol (reference), and (b) PL spectra of Eu-doped strontium aluminate (x = 3) and ethanol, yielding AQYs of ~3.3% and ~33.4%, respectively (see **Table S2** for the excitation/emission wavelengths used).

Table S6. Comparison of emission peaks and AQYs of various Eu^{2+} or Eu^{3+}-doped phosphors from the literature vs. this work. The Eu-doped strontium aluminate phosphors developed in this work exhibit competitive or superior quantum yields compared to other reported materials.

Phosphor	Emission peak (nm)	AQY	Ref.
$Na_2(Ba,Gd)_6(Si_2O_7)(SiO_4)_2$	445	12%	[2]
$Ca_{0.9995}S:Eu_{0.0005}^{2+}$	640	32%	[3]
$CsSrCl_3:Eu^{2+}$	430	40%	[4]
**$SrAl_2O_4$:2%Eu^{2+}**	**520**	**46.5%**	**This work**
$Ba_6Lu_{4.6}B_9O_{27}:Eu_{0.4}^{3+}$	621	28%	[5]
$Ca_2NaMg_2V_3O_{12}:Eu^{3+}$	512	45%	[6]
$Ca_{10}(PO_4)_6F_2:Eu^{3+}$	618	62.8%	[7]
**$SrAl_1O$:2%Eu^{3+}**(x=Al/Sr=1)	**595**	**66.2%**	**This work**

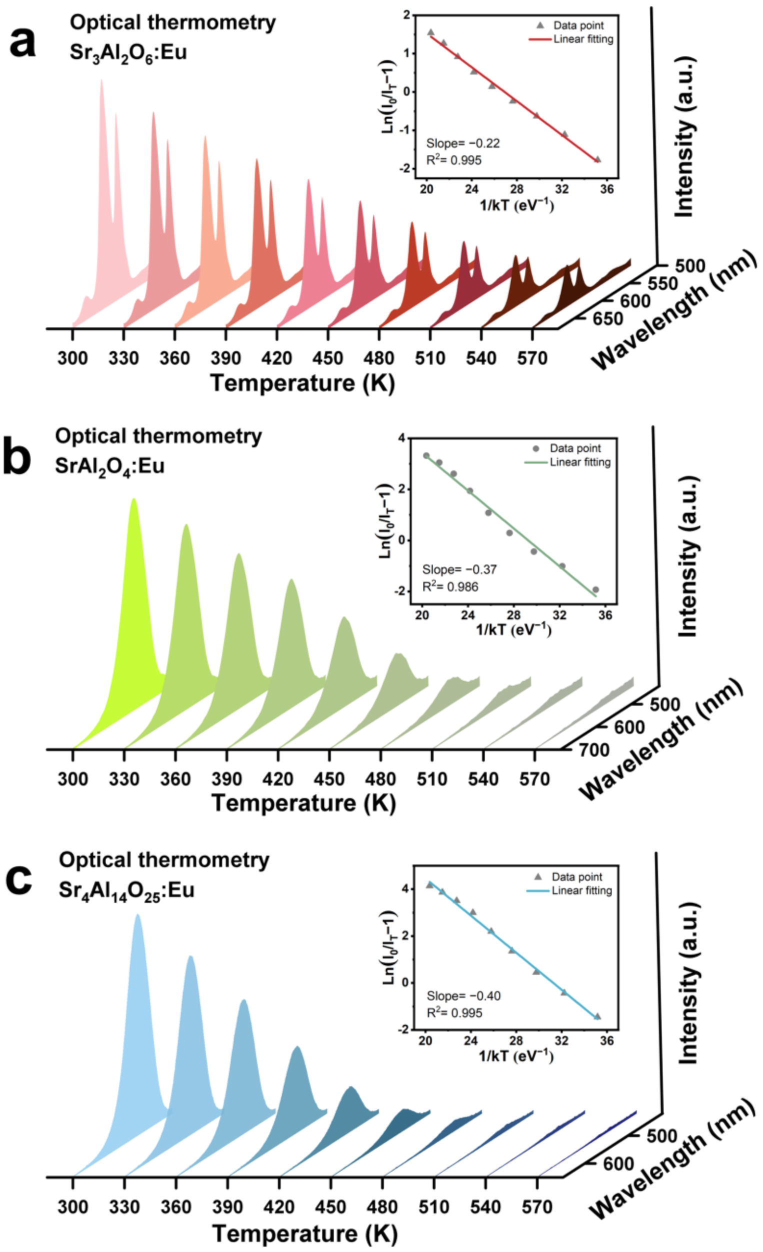

Figure S13. Temperature-dependent PL spectra (λ_{ex} = 254 nm) for (a) $Sr_3Al_2O_6$:Eu, (b) $SrAl_2O_4$:Eu, and (c) $Sr_4Al_{14}O_{25}$:Eu from 300 K to 570 K. Insets: Arrhenius plots [ln$(I_0/I_T - 1)$ vs 1/kT] used to extract thermal activation energies E_a. Calculated E_a: 0.22 eV (a), 0.37 eV (b) and 0.40 eV (c). Higher E_a corresponds to stronger quenching (the Al-rich phases quench more strongly, indicating a defect-driven quenching mechanism).

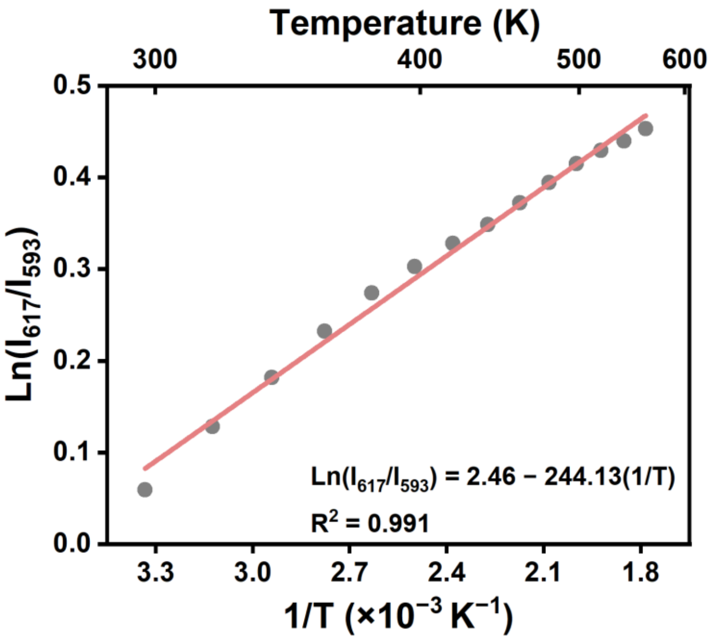

Figure S14. Plot of Ln(I_{617}/I_{593}) vs 1/T for Eu-doped strontium aluminate (x = 1) phosphors, showing a linear fit Ln(I_{617}/I_{593}) = 2.46 – 244.13 (1/T) ($R^2 \approx 0.992$). This linear relationship underpins the luminescent thermometry calibration for this phosphor.

Table S7. Summarized maximum relative sensitivity (Max. S_r) previously reported for Lanthanide-doped materials.

**Materials**	**Max. S_r (% K^{-1})**	**Temperature range (K)**	**Ref.**
$GdVO_4:Sm^{3+}$	0.045	293-823	[8]
$Gd_2O_3:Eu^{3+}$	0.07	300-800	[9]
Er^{3+} doped silicate glasses	0.23	296-673	[10]
$Ca_2LaNbO_6:Sm^{3+}$	0.22	313-573	[11]
(65-x)TeO_2-20ZnF_2-12PbO-3Nb_2O_5-$x$$Sm_2O_3$	0.23	300-700	[12]
**$SrAl_1O:Eu^{3+}$(x = Al/Sr = 1)**	**0.27**	**300-560**	**This work**

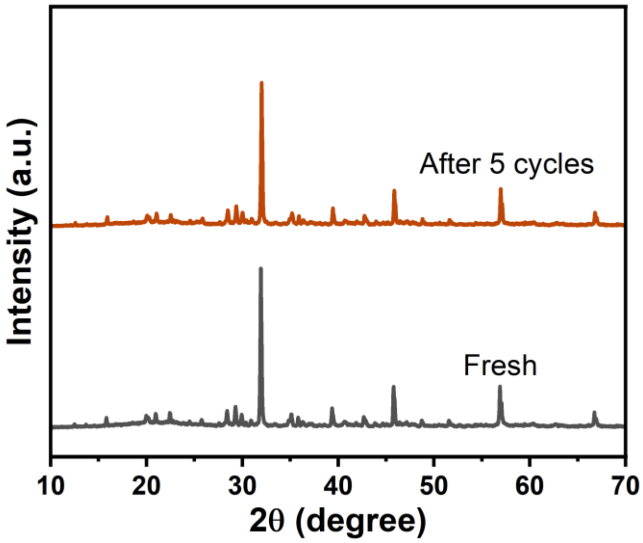

Figure S15. XRD patterns of Eu-doped strontium aluminate ($x = 1$) phosphors before and after five thermal cycles between 300 K and 560 K. The coincidence of peaks before vs. after indicates no detectable structural degradation or phase change, confirming excellent thermal stability of the host lattice under repeated thermal stress.

References

1. Vitola, V.; Millers, D.; Bite, I.; Smits, K.; Spustaka, A. Recent Progress in Understanding the Persistent Luminescence in $SrAl_2O_4$:Eu,Dy. *Mater. Sci. Technol.* **2019**, *35*, 1661–1677.
2. Zhu, Y.; Liang, Y.; Liu, S.; Wu, X.; Xu, R.; Li, K. Structural Evolution Induced Preferential Occupancy of Eu^{2+} in $Na_2(Ba,Gd)_6(Si_2O_7)(SiO_4)_2$ Phosphor. *Mater. Res. Bull.* **2017**, *90*, 47–50.
3. Katumo, N.; Li, K.; Richards, B.S.; Howard, I.A. Dual-Color Dynamic Anti-Counterfeiting Labels with Persistent Emission after Visible Excitation Allowing Smartphone Authentication. *Sci. Rep.* **2022**, *12*, 2100.
4. Lei, C.; Wu, X.; Li, Y.; Xu, X.; Zuo, G.; Ou, Q.; Zhang, S. Eu-Doped $CsSrCl_3$ Large Nanocrystal Clusters with Self-Reduction Effect and near-Unity Quantum Yield. *Laser*

Photonics Rev. **2025**, e01672.

5. Bubnova, R.S.; Povolotskiy, A.V.; Biryukov, Y.P.; Kolesnikov, I.E.; Volkov, S.N.; Filatov, S.K. Cation Sites Occupation and Luminescence of Novel Red-Emitting Phosphors $Ba_6(Lu_{1-x}Eu_x)_5B_9O_{27}$ (x = 0.02–0.2). *Ceram. Int.* **2022**, *48*, 15966–15974.
6. Pasinski, D.; Sokolnicki, J. Nitridated $Ca_2NaMg_2V_3O_{12}$: Eu^{3+} Vanadate Garnet Phosphor-in-Glass. *Materials* **2020**, *13*, 2996.
7. Bie, S.-R.; She, D.-S.; Yue, W. Luminescence Mechanism and Optical Properties of Apatite-Type $Ca_{10}(PO_4)_6F_2$: Eu^{3+} Phosphors. *J. Lumin.* **2025**, *281*, 121173.
8. Nikolic, M.G.; Jovanovic, D.J.; Dordevic, V.; Antic, Z.; Krsmanovic, R.M.; Dramicanin, M.D. Thermographic Properties of Sm^{3+}-Doped $GdVO_4$ Phosphor. *Phys. Scr.* **2012**, *T149*.
9. Nikolić, M.G.; Al-Juboori, A.Z.; Đorđević, V.; Dramićanin, M.D. Temperature Luminescence Properties of Eu^{3+}-Doped Gd_2O_3 Phosphors. *Phys. Scr.* **2013**, *2013*, 14056.
10. Li, C.; Dong; B.; Ming, C.; Lei, M. Application to Temperature Sensor Based on Green Up-Conversion of Er^{3+} Doped Silicate Glass. *Sensors*. **2007**, *7*, 2652-2659.
11. Zhang, A.; Sun, Z.; Jia, M.; Fu, Z.; Choi, B.C.; Jeong, J.H.; Park, S.H. Sm^{3+}-Doped Niobate Orange-Red Phosphors with a Double-Perovskite Structure for Plant Cultivation and Temperature Sensing. *J. Alloys Compd.* **2021**, *889*, 161671.
12. Klimesz, B.; Lisiecki, R.; Ryba-Romanowski, W. Sm^{3+}-Doped Oxyfluorotellurite Glasses - Spectroscopic, Luminescence and Temperature Sensor Properties. *J. Alloys Compd.* **2019**, *788*, 658–665.
